# Reduced SuM Activation Accompanies Impaired Social Novelty Recognition in Mouse Models of Neurodevelopmental Disorders

**DOI:** 10.1523/ENEURO.0440-25.2026

**Published:** 2026-07-07

**Authors:** Raymond Maejima, Yuki Ito, Jun Motoyama

**Affiliations:** ^1^Laboratory of Developmental Neurobiology, Graduate School of Brain Science, Doshisha University, Kyotanabe, Kyoto 610-0394, Japan; ^2^Organization of Advanced Research and Education, Doshisha University, Kyotanabe, Kyoto 610-0394, Japan; ^3^Centre for Baby Science, Doshisha University, Kyotanabe, Kyoto 610-0394, Japan

**Keywords:** Caps2 deficiency, GPR54, maternal separation, mouse, social novelty recognition, SuM

## Abstract

Social novelty recognition—the ability to distinguish between familiar and unfamiliar individuals—is frequently disrupted in neurodevelopmental disorders, including those resulting from early-life stress and autism spectrum disorder (ASD). The supramammillary nucleus (SuM) has been implicated as a key region involved in processing novelty-related information and associated with hippocampal CA2 activity; however, whether SuM dysfunction occurs in neurodevelopmental disorder models with impaired social novelty recognition remains unclear. Here, we examined c-Fos expression in the SuM, CA2, and dentate gyrus (DG) following exposure to a novel conspecific in male mice from two models: maternal separation (MS) mice representing early-life stress and *Caps2^−/−^* mice, a genetic model of ASD. Novel social encounters robustly induced c-Fos expression in the SuM, dorsal and ventral CA2, as well as dorsal and ventral DG regions in control mice, whereas such induction was not observed in either MS or *Caps2^−/−^* mice. Furthermore, GPR54 expression in the SuM differed between the two models, with MS mice showing reduced expression, whereas *Caps2^−/−^* mice exhibited an increased number of GPR54-expressing cells. These findings suggest that reduced activation of the SuM, CA2, and DG is a shared feature across distinct models with impaired social novelty recognition. Furthermore, the divergent patterns of GPR54 expression raise the possibility that KISS1–GPR54 signaling may differentially modulate SuM function across models, although its causal role remains to be determined.

## Significance Statement

We examined whether the supramammillary nucleus (SuM) is altered in mouse models with impaired social novelty recognition. In control mice, exposure to a novel conspecific robustly induced c-Fos expression in the SuM, hippocampal CA2, and dentate gyrus (DG), whereas maternal separation (MS) and *Caps2^−/−^* mice showed reduced activation in these regions. The two models also exhibited divergent changes in SuM GPR54 expression, with reduced expression in MS mice and increased numbers of GPR54-expressing cells in *Caps2^−/−^* mice. These findings suggest that impaired social novelty recognition is associated with reduced SuM, CA2, and DG activation across distinct models and that altered GPR54 expression may reflect model-specific molecular abnormalities linked to different etiologies.

## Introduction

Social novelty recognition, the ability to distinguish unfamiliar from familiar conspecifics, is a fundamental component of mammalian social behavior. This capacity contributes to social adaptation, organization of group structure, and ultimately survival and reproductive success. Disruptions in social novelty recognition are commonly observed in neurodevelopmental disorders, including those associated with excessive early-life stress and autism spectrum disorder (ASD). These observations suggest that neural mechanisms underlying this behavior emerge during fetal and postnatal development. Neonatal stress models, such as maternal separation (MS), exhibit deficits in social novelty recognition along with long-lasting alterations in neurogenesis, stress reactivity, and connectivity within limbic and hypothalamic circuits (Naninck et al., 2015; Rombaut et al., 2023). Likewise, ASD is associated with abnormal prenatal and postnatal neural development, including excitatory–inhibitory imbalance, atypical synaptic pruning, and impaired neuromodulatory signaling (Banerjee et al., 2014; Ellegood and Crawley, 2015; Kaushik and Zarbalis, 2016; Chen and Hong, 2018). Although MS and ASD model mice each recapitulate key aspects of impaired social behavior, the neural bases responsible for social novelty deficits remain incompletely understood.

Social novelty recognition engages a distributed network of brain regions. The medial amygdala and bed nucleus of the stria terminalis receive olfactory and pheromonal inputs from the accessory olfactory bulb and relay socially relevant cues to higher brain regions (Choi et al., 2005). The hippocampus and medial prefrontal cortex contribute to the encoding and retrieval of social memory, providing contextual and temporal frameworks for social encounters (Hitti and Siegelbaum, 2014; Okuyama et al., 2016). The lateral septum and several hypothalamic nuclei including the paraventricular nucleus and supramammillary nucleus (SuM) further modulate social and affective responses (Chen et al., 2020; Rizzi-Wise and Wang, 2021; Thirtamara Rajamani et al., 2024). Among these regions, SuM has emerged as a critical hub linking hypothalamic, septal, and hippocampal circuits (Pan and McNaughton, 2004). Anatomical studies in rodents have demonstrated that the SuM receives inputs from the medial septum, nucleus accumbens, some hypothalamic regions, and brainstem arousal centers and sends prominent excitatory projections to the hippocampus as well as to the medial septum and thalamic targets (Wyss et al., 1979; Maglóczky et al., 1994; Vertes, 2015). Consistent with the anatomical connectivity between the hippocampus and the SuM, medial SuM (mSuM) lesions in rats induce motivational and emotional behavioral changes—such as hyperactivity in defensive and operant tasks—that resemble those observed following hippocampal lesions. In contrast, the mSuM lesions do not affect spatial learning in the water maze (Pan and McNaughton, 2002). Recent findings highlight its function in novelty processing: SuM neurons projecting to CA2 preferentially respond to social novelty, whereas those projecting to the dentate gyrus (DG) encode contextual novelty (Chen et al., 2020). Despite this evidence, the impact of mSuM lesions on social behaviors has not been examined, and it remains unknown whether the SuM is activated in response to social novelty in MS or ASD model mice that exhibit impairments in social novelty recognition.

Social novelty recognition emerges as early as postnatal day 14 (P14; Diethorn and Gould, 2022). Neural activity within the hippocampal CA2 region is essential for this behavior, and projections from the SuM to CA2 are already established by P14 (Diethorn and Gould, 2022). At the molecular level, the kisspeptin (KISS1) receptor GPR54—a molecular marker for a major subset of SuM neurons—begins to be expressed approximately P30 and increases into adulthood, as shown in *Gpr54-lacZ* reporter mice (Herbison et al., 2010; Higo et al., 2016). KISS1 is classically known for regulating the pubertal onset and triggering reproductive hormone release through activation of hypothalamic GnRH neurons (de Roux et al., 2003; Seminara et al., 2003). Although KISS1–GPR54 signaling is primarily associated with reproductive maturation, its intracellular actions enhance neuronal excitability and synaptic plasticity (Kotani et al., 2001; Liu et al., 2008), suggesting a potential contribution to the pubertal maturation of circuits supporting the establishment of social novelty recognition. While *Kiss1-* or *Gpr54*-deficient mice exhibit marked reproductive and sexual behavioral abnormalities, possible roles in nonreproductive social behaviors remain unclear (Kauffman et al., 2007; Lapatto et al., 2007; Clarkson et al., 2008; Chan et al., 2009). It has not yet been determined whether the number or expression levels of GPR54-expressing cells in the SuM is altered in MS or ASD model mice with impaired social novelty recognition.

Here, we examined the functional contribution of the SuM to social novelty recognition across development and in two neurodevelopmental disorder models with distinct etiologies. We first assessed developmental changes in GPR54 expression in the SuM from puberty to adulthood and examined age-dependent changes in social novelty recognition. We then tested whether SuM lesions during puberty affect adult social novelty recognition. In addition, we assessed c-Fos expression in the SuM and hippocampal CA2 and DG following exposure to a novel conspecific in each model. Finally, we evaluated GPR54 expression across these disorder models. To represent distinct etiological backgrounds, we used MS mice, modeling early-life stress, and calcium-dependent activator protein for secretion 2 (CAPS2)-deficient (*Caps2^−/−^*) mice, a genetic model exhibiting ASD-related phenotypes. MS mice, which underwent chronic maternal separation from P2 to P21, exhibit abnormal social behavior, long-lasting alterations in stress reactivity, and changes in hippocampal plasticity, thereby modeling the impact of early-life stress on neurodevelopment (Liu et al., 1997; O'Mahony et al., 2011). In contrast, CAPS2 plays a critical role in neuropeptide release at presynaptic terminals, axonal transport, and priming of dense-core vesicles, and *Caps2^−/−^* mice exhibit impaired social behavior, hyperactivity, reduced exploratory behavior, and increased anxiety in novel environments, reflecting core ASD-like features (Sadakata et al., 2007; Shinoda et al., 2011). These approaches allowed us to examine convergent patterns of circuit-level alterations alongside potential model-specific molecular differences.

## Materials and Methods

### Mice

All experiments were performed in accordance with the Guidelines for the Care and Use of Laboratory Animals of the Animal Research Committee of Doshisha University (license number: A25060, A25067). All mice were on a C57BL/6 background, and only male mice were used. Wild-type C57BL/6 mice were obtained from Shimizu Laboratory Supplies. Maternal separation (MS) was conducted as previously described with minor modifications (Shin et al., 2023). From P2 to P21, pups were separated from the dam for 4 h daily (11:00–15:00). Only male pups were separated from the dam, whereas female pups remained in the home cage during the separation period. This partial-litter separation design was adopted based on previous studies aiming to minimize excessive disruption of maternal care caused by complete litter removal while still inducing early-life social stress in male pups. During separation, pups were placed in a clean, temperature-controlled cage (34–36°C) containing bedding to maintain body temperature. Control litters remained undisturbed with the dam except for routine husbandry procedures. After weaning at P22, MS and control mice were group housed separately until testing. CAPS2-deficient (*Caps2^−/−^*) mice were kindly provided by Prof. Furuichi (RBRC02949, RIKEN BRC; Sadakata et al., 2012). Homozygous mutants were generated by mating *Caps2^+/−^* females with *Caps2^−/−^* males, and the genotypes of their offspring were confirmed by PCR following the RIKEN BRC protocol. Mice were group housed (3–5 per cage) in a temperature- and humidity-controlled room (23 ± 2°C; 50 ± 20% relative humidity) under a 12 h light/dark cycle (lights on at 08:00, off at 20:00), with *ad libitum* access to food and water. Assignment to experimental groups was based on genotype or maternal separation condition. Data collection and analysis were conducted without blinding.

### Ibotenic acid injection

The mice were anesthetized with avertin at P35 (100 mg/kg of body weight, i.p.) and positioned in a stereotaxic frame (Model 902, KOPF Instruments). Then, 4 µg/µl of ibotenic acid (IBA) solution (12765, Sigma-Aldrich) was delivered into the SuM using a pulled-glass pipette (Model G1, Narishige Scientific Instrument Lab). Coordinates relative to bregma were A/P −2.50 mm, M/L 0.0 mm, D/V −5.00 mm. Then, 50–100 nl of IBA solution was injected using an electric microinjector (Model BJ-120, BEX). Controls received PBS injections. Mice recovered on a heating pad before being returned to their home cages. Behavioral testing was conducted 2 weeks later (P49). Lesions caused by injected IBA solution were confirmed by NeuN staining.

### Sample preparation and immunohistochemistry

Mice were deeply anesthetized and transcardially perfused with phosphate-buffered saline (PBS), pH 7.4, followed by 4% paraformaldehyde (PFA, Nacalai Tesque) in PBS. Brains were postfixed in 4% PFA overnight at 4°C, dehydrated through graded ethanol, cleared in xylene, and embedded in paraffin (#101676, Histosec 60, Sigma-Aldrich). Coronal sections (10 μm thick) were cut using a rotary microtome (RM2125, Leica) and mounted on glass slides (Matsunami). For immunohistochemistry, sections were deparaffinized, rehydrated, and subjected to antigen retrieval in 10 mM citrate buffer, pH 6.0, at 95°C (three times for 15 min). After cooling, sections were blocked for 1 h at room temperature in PBS containing 1 mg/ml BSA, 2% gelatin, 1% Triton X-100, and 0.02% sodium azide. Primary antibodies were applied overnight at 4°C: rabbit anti-GPR54 (1:100, AKR-001, Alomone Labs), rabbit anti-NeuN (1:500, ABN78, Millipore), rat anti-c-Fos (1:200, 226017, Synaptic Systems), and guinea pig anti-PCP4 (1:500, 480004, Synaptic Systems). Following PBS washes, sections were incubated for 1 h with the appropriate secondary antibodies: goat anti-rabbit Alexa Fluor 488 (1:200, A11034, Invitrogen) or goat anti-rat Alexa Fluor 568 (1:200, A11081, Invitrogen). Nuclei were counterstained with Hoechst 33342 (1:400, Nacalai Tesque). Slides were coverslipped with Immu-Mount (#9990412, Epredia), and images were acquired using an Olympus BX51 fluorescence microscope equipped with a DP30BW camera. Negative controls processed without primary antibodies showed no positive signal.

### Cell quantification

For developmental analysis, GPR54-expressing cells were quantified at three anteroposterior (A/P) levels of the SuM (A/P −2.54, −2.80, and −3.08 mm from bregma). Three coronal sections per mouse were analyzed (*n* = 3 mice per age group). GPR54-expressing cells were counted in five subregions within the ventral SuM (left distal, left proximal, medial, right proximal, and right distal), each defined as a 0.145 mm^2^ region of interest (Extended Data Fig. 1-1). Mean GPR54-expressing cell density was calculated from 15 regions per mouse (5 subregions × 3 sections).

For the MS and *Caps2^−/−^* experiments, GPR54-expressing cells were quantified in three 0.145 mm² subregions within the left lateral SuM (SuM_LL_), medial SuM (SuMm), and right lateral SuM (SuM_RL_) at A/P −2.54 mm, −2.80 mm, and −3.08 mm from bregma (Extended Data Fig. 5-1). Three coronal sections per mouse were analyzed (*n* = 3 mice per group). Mean GPR54-expressing cell density for each mouse was calculated by averaging values across the three sections.

To assess lesion extent, NeuN^+^ cells were counted in a 0.145 mm^2^ region within the ventromedial SuM at A/P −2.80 mm from bregma using one coronal section per mouse at P49 (*n* = 3 per group). Mean NeuN^+^ cell numbers were compared between PBS- and IBA-injected mice.

c-Fos-positive cells in the SuM were quantified at A/P −2.54 mm, −2.80 mm, and −3.08 mm from bregma in three subregions using three coronal sections per mouse (*n* = 3 mice per group; Extended Data Fig. 5-1). For c-Fos-positive cell quantification in the hippocampus, three stitched composite images were generated per mouse by combining tiled images for each section: 2 tiles for the dorsal CA2 (A/P: −1.94 mm from bregma) and ventral CA2 (A/P: −2.54 mm from bregma), 6–7 tiles for the dorsal DG (A/P: −1.94 mm from bregma), and 5–6 tiles for the ventral DG (A/P: −2.54 mm from bregma). The entire CA2 region was defined based on PCP4 expression, and the PCP4-expressing pyramidal cell layer was traced using ImageJ. The boundaries of the DG regions were defined according to Paxinos and Franklin (2013) and traced using ImageJ. c-Fos^+^ cells containing Hoechst-positive nuclei were counted within the traced areas. For each section, cell density was calculated by dividing the mean number of c-Fos^+^ cells across the left and right hemispheres by the corresponding mean surface area. These values were then averaged across the three composite images to determine the mean cell density for each mouse (*n* = 3 mice per group).

### Relative fluorescence intensity of GPR54

GPR54 fluorescence intensity was measured in each GPR54-expressing cell colocalized with Hoechst^+^ nuclei while excluding the nuclear region. ROIs were manually created on a cell-by-cell basis in ImageJ. No background subtraction was applied. The measurements were taken from the medial region at A/P −2.80 mm in three sections per mouse (*n* = 3 per group). The distributions of fluorescence intensity for each GPR54-expressing cell are presented descriptively without statistical testing.

### Behavioral analyses

The three-chamber test followed established protocols (Rein et al., 2020). Interaction time was defined as the duration during which the test mouse stayed within 10 cm of the edge of each chamber containing either the novel mouse (S1 or S2) or the object. Discrimination indices were calculated as: Sociability: (S1 − object) / (S1 + object) and Social novelty: (S2 − S1) / (S2 + S1). After the three-chamber test, direct social interaction was assessed in an open field (45 × 45 × 45 cm). Mice were habituated for 15 min/d for 10 d (Chen et al., 2020). On Day 11, a novel conspecific male was introduced first, followed by the test mouse, and interaction lasted 5 min. Social interaction was quantified as the total duration of sniffing behavior of the test mouse directed toward the stimulus mouse. Sniffing behavior was defined as the test mouse's nose being within ∼2.5 cm of the stimulus mouse's nose (facial sniffing), body (body sniffing), or anogenital region (anogenital sniffing). The behavior of the test mice in both behavioral analyses was recorded with an overhead video camera (Logicool HD Pro Webcam C920, Logitech) and analyzed using UMATracker and DeepLabCut (Mathis et al., 2018; Yamanaka and Takeuchi, 2018).

### Social novelty exposure for c-Fos detection

To more rigorously evaluate c-Fos induction associated with social novelty exposure across all three groups (control, MS, and *Caps2^−/−^*), we included matched control groups without social novelty exposure. These control animals underwent the same experimental procedures as the social novelty exposure group. Specifically, all mice underwent 10 d of habituation to the test arena beginning at P57 while remaining group housed. Immediately after the habituation session on Day 10, mice were single housed to minimize background c-Fos expression associated with social interactions prior to testing. On Day 11, experimental mice were exposed to a novel mouse, whereas control mice were individually placed in the same arena for an identical duration without a stimulus mouse. Brains were collected 1.5 h after the end of the social exposure session for c-Fos immunohistochemistry.

### Statistical analysis

Statistical analyses were performed using GraphPad Prism 10 (GraphPad Software). Data are presented as mean ± SEM. Detailed statistical information, including groups/factors, data structure, statistical tests, test statistics, post hoc analyses, *p* values, and sample sizes (*n* per group) for all figures are provided in Extended Data Figure 1-2. Normality was assessed using the Shapiro–Wilk test, and variance homogeneity using the Brown–Forsythe test. Two-way repeated-measures ANOVA with Šídák post hoc tests was used for within-group comparisons in the three-chamber test, direct social interaction test, and cell quantification of c-Fos-positive and c-Fos/GPR54-expressing cells. Between-group comparisons were performed using unpaired Welch's *t* tests or one-way ANOVA (with Tukey's test), depending on the number of groups. Nonparametric tests (Kruskal–Wallis with Dunn’s test; multiple Mann–Whitney with Holm–Šídák) were used when normality was not met. When data failed the assumption of equal variances, Welch’s one-way ANOVA followed by Dunnett's T3 test was used; significance was set at *p* < 0.05.

### Figure preparation

Schematic illustrations were generated using BioRender. Image processing was conducted using ImageJ (NIH), Adobe Photoshop (Adobe Systems), and GIMP (GNU Image Manipulation Program). Figures were assembled in Microsoft PowerPoint (Microsoft Corporation).

## Results

### Developmental increase in GPR54-expressing cells in the SuM between P35 and P56

**Figure 1.**
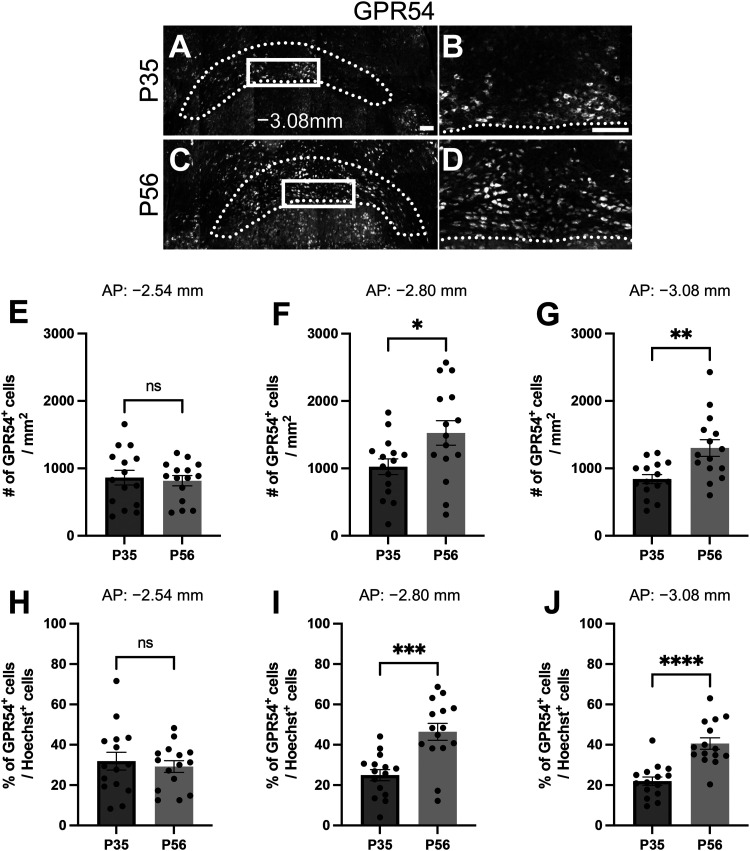
Increased GPR54 expression in the SuM from P35 to P56. ***A–D***, Images of GPR54-expressing cells in the SuM detected by using immunohistochemistry and observed at lower (***A***, ***C***) and higher magnification (***B***, ***D***) at P35 (***A***, ***B***) and P56 (***C***, ***D***). Dotted lines in ***A*** and ***C*** outline the SuM at A/P: −3.08 mm from bregma. The enlarged images of the rectangles in ***A*** and ***C*** are ***B*** and ***D***, respectively. Scale bars in ***A*** and ***B*** are 100 μm. The scale bar in ***A*** is for ***A*** and ***C*** and that in ***B*** is for ***B*** and ***D***. ***E–G***, The density of GPR54-expressing cells in three different locations (***E***, A/P −2.54 mm; ***F***, −2.80 mm; ***G***, −3.08 mm) in the SuM on the anterior–posterior axis at P35 and P56. GPR54-expressing cells were counted in five subregions within the ventral SuM (left distal, left proximal, medial, right proximal, and right distal), each defined as a 0.145 mm^2^ region of interest (Extended Data Fig. 1-1). The number of GPR54-expressing cells per mm^2^ was compared between P35 and P56. ***H–J***, The percentage of GPR54-expressing cells among Hoechst^+^ cells in the SuM at three different locations per mm^2^ at P35 and P56. Filled circles in ***E–J*** represent individual values from the five SuM subregions in each section. All values are represented as mean ± SEM in ***E–J***. *p < 0.05, **p < 0.01, ***p < 0.001, ****p < 0.0001, ns, not significant. See Extended Data Figure 1-2 for detailed statistical information.

Previous work using *Gpr54-lacZ* reporter mice indicated that GPR54 expression begins approximately P30 and increases into adulthood (Herbison et al., 2010). To characterize the developmental change of GPR54 protein expression in the SuM from puberty to adulthood, we performed immunohistochemistry in three different locations (A/P −2.54 mm, −2.80 mm, and −3.08 mm) on the anterior–posterior axis at P35 and P56 and quantified GPR54-expressing cells across five anatomically defined SuM subregions (Fig. 1*A–G*; Extended Data Fig. 1-1). An increase in the density of GPR54-expressing cells was observed in the SuM at A/P −2.80 mm and −3.08 mm (Fig. 1*F*,*G*). GPR54-expressing cell density increased from 1,025 ± 115.7 cells/mm^2^ at P35 to 1,526 ± 181.9 cells/mm^2^ at P56 at A/P −2.80 mm and from 841.9 ± 67.55 cells/mm^2^ at P35 to 1,303 ± 124.2 cells/mm^2^ at P56 at A/P −3.08 mm, representing approximately a 1.5-fold increase (Fig. 1*F*,*G*). Likewise, the proportion of GPR54-expressing cells within the SuM nearly doubled from 24.95 ± 2.77% at P35 to 46.43 ± 4.2% at P56, representing approximately a 1.8-fold increase (Fig. 1*I*,*J*). No obvious difference in the density and the proportion of GPR54-expressing cells within the SuM was observed in the anterior SuM at A/P −2.54 mm from bregma (Fig. 1*E*,*H*). These results indicate that the number of GPR54-expressing cells increases from puberty to adulthood.

10.1523/ENEURO.0440-25.2026.f1-1 Figure 1-1 Schematic diagram of the mouse coronal section indicating the position and outline of SuM at A/P: −2.54, −2.80, and −3.08 mm from bregma. The five frames in the SuM indicate the subregions where GPR54⁺ cells were counted at P35 and P56. Download Figure 1-1, TIF file.

10.1523/ENEURO.0440-25.2026.f1-2 Figure 1-2 Statistical analyses supporting Figures 1–8 and associated Extended Data figures. Download Figure 1-2, TIF file.

### Development of social novelty recognition from P35 to P56

**Figure 2.**
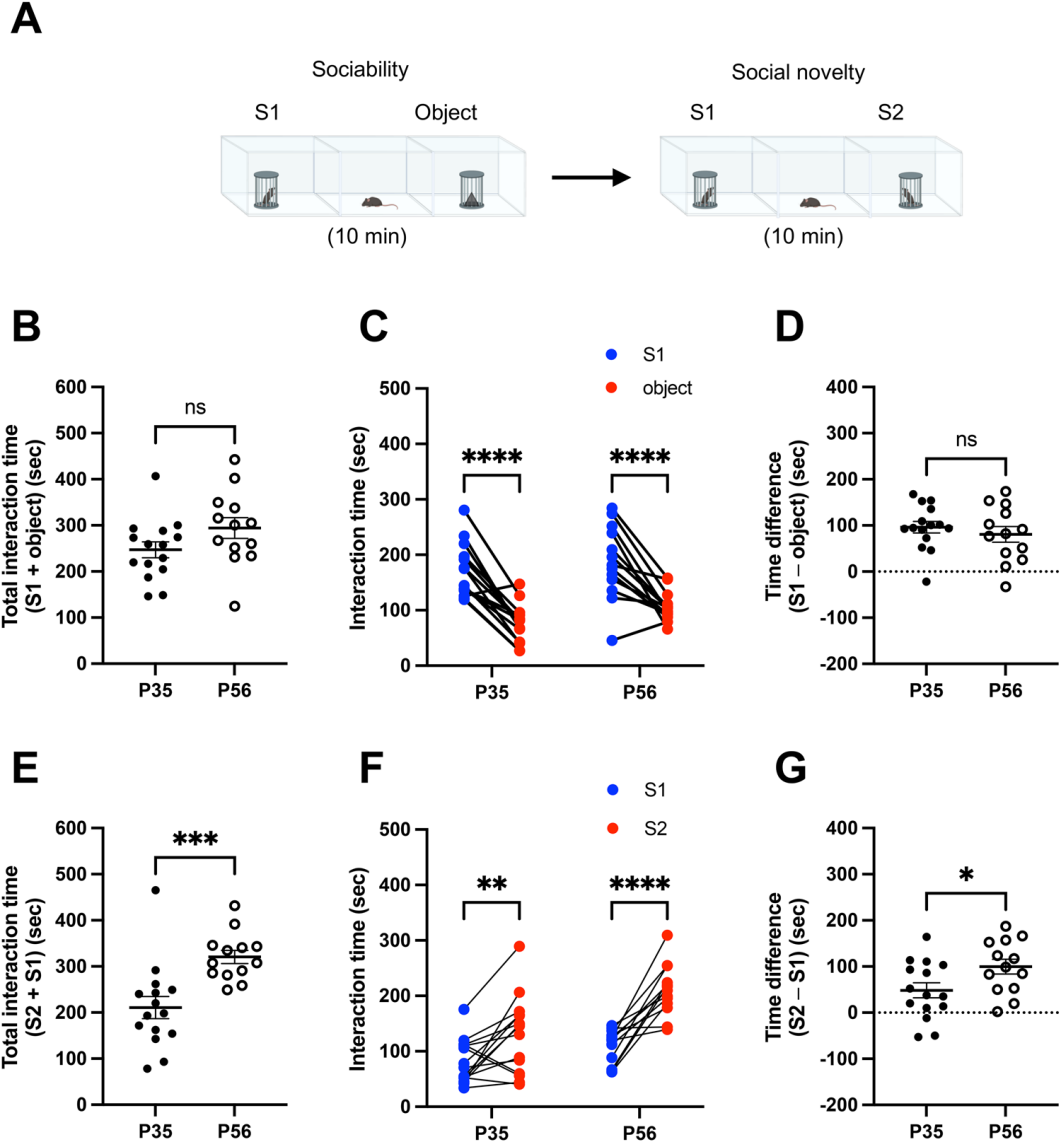
Temporal change of social recognition between P35 and P56. ***A***, Schematic of the three-chamber test apparatus and testing procedure. In the sociability test, a novel mouse (S1) was placed in one chamber, while the other chamber contained a novel object (object). In the social novelty test, the object was replaced by a new novel mouse (S2), while S1 remained in the same chamber. The behavior of the test mouse was examined for 10 min. ***B***, Total interaction time (S1 + object) of the test mouse at P35 and P56. ***C***, Within-group comparisons of the test mouse's interaction time with S1 and the object at P35 and P56. ***D***, Difference in interaction time with S1 versus the object (S1 − object) between P35 and P56. See Extended Data Figure 2-1 for the sociability discrimination index analysis. ***E***, Total interaction time (S1 + S2) of the test mouse at P35 and P56. ***F***, Within-group comparisons of the test mouse's interaction time with S1 and S2 at P35 and P56. ***G***, Difference in interaction time with S1 versus S2 (S2 − S1) between P35 and P56. P56 mice showed a markedly larger difference in interaction time between S1 and S2. See Extended Data Figure 2-2 for the social novelty discrimination index analysis. Filled and unfilled circles in ***B***, ***D***, ***E***, and ***G*** represent data from individual mice. In ***C*** and ***F***, filled blue and red circles represent interaction times of individual mice (blue, S1; red, object in ***C***; and blue, S1; red, S2 in ***F***), and connecting lines between filled blue and red circles represent the same mouse. All values are represented as mean ± SEM in ***B***, ***D***, ***E***, and ***G***. *p < 0.05, **p < 0.01, ***p < 0.001, ****p < 0.0001, ns, not significant. See Extended Data Figure 1-2 for detailed statistical information.

Given the developmental increase in SuM GPR54 expression, we examined whether social novelty recognition improves between P35 and P56 using three-chamber tests (Fig. 2*A*). Sociability did not differ between age groups: total interaction time with S1 + object was comparable (P35: 247 ± 17.04 s; P56: 294 ± 22.56 s), and both groups showed significantly more interaction with S1 than with the object (Fig. 2*B–D*; Extended Data Fig. 2-1). In the social novelty test, total interaction time (S1 + S2) was significantly longer at P56 (320.6 ± 14.35 s) compared with P35 (210.9 ± 23.99 s; Fig. 2*E*). Although both groups preferred the novel conspecific (S2) over the familiar mouse (S1), P56 mice showed a markedly larger difference in interaction time (P35: 48.38 ± 16.22 s; P56: 99.61 ± 16.04 s; Fig. 2*F*,*G*; Extended Data Fig. 2-2). Thus, the ability to discriminate novel from familiar conspecifics is already present by P35, but social engagement and exploration toward the novel mouse increase substantially by P56. This developmental enhancement parallels the observed increase in SuM GPR54 expression.

10.1523/ENEURO.0440-25.2026.f2-1 Figure 2-1 Discrimination index for the sociability phase at P35 and P56 mice. P35 mice exhibited a stronger preference for S1 than P56 mice (*n* = 15 mice for P35, 13 mice for P56). *p < 0.05. See Figure 1-2 for detailed statistical information. Download Figure 2-1, TIF file.

10.1523/ENEURO.0440-25.2026.f2-2 Figure 2-2 Discrimination index for the social novelty phase at P35 and P56 mice. No significant difference of the preference toward S2 between P35 and P56 mice (*n* = 15 mice for P35, 13 mice for P56). ns, not significant. See Figure 1-2 for detailed statistical information. Download Figure 2-2, TIF file.

### P35 SuM lesions impair adult social novelty recognition

**Figure 3.**
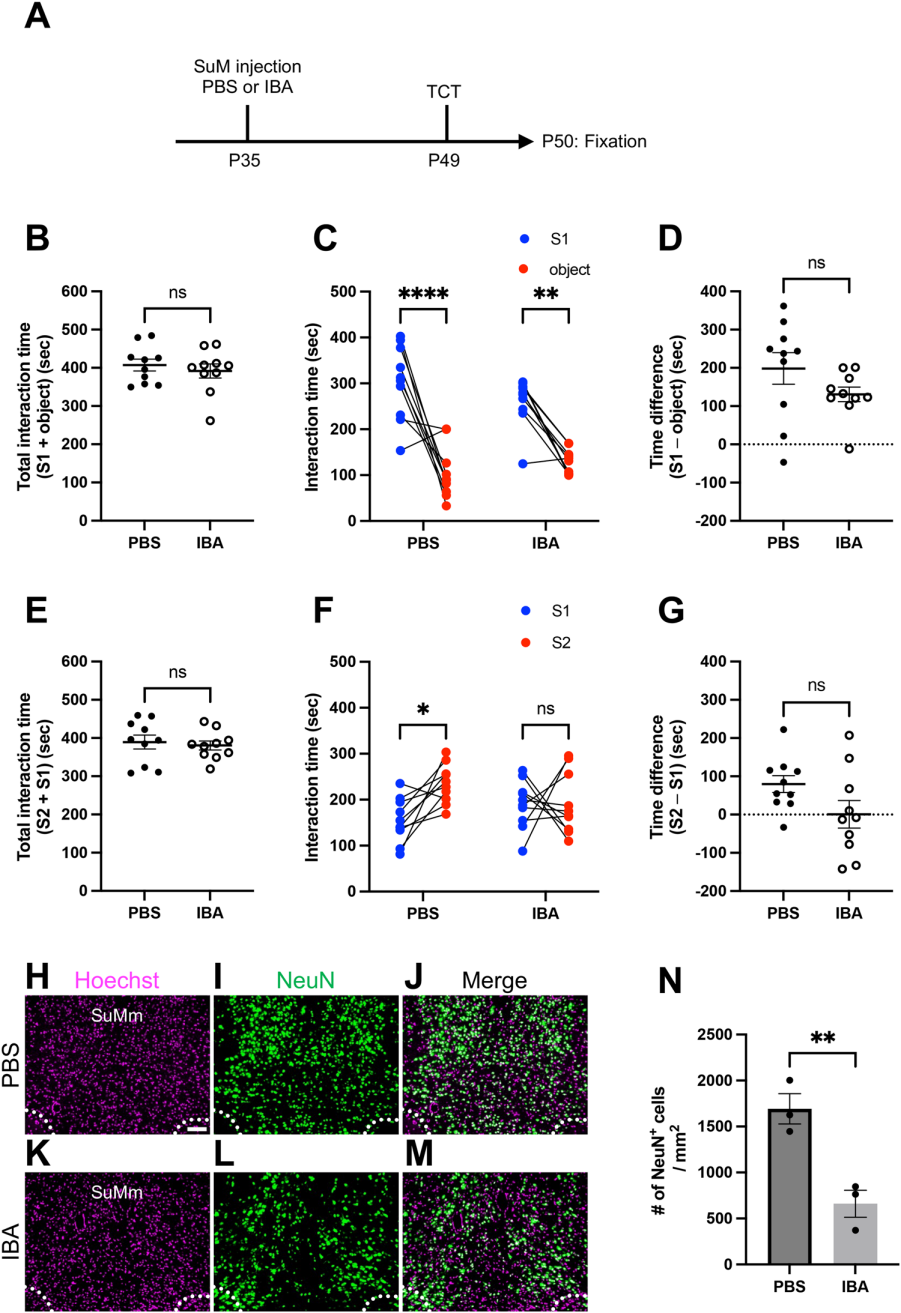
Lesioning the SuM by IBA injection impaired social novelty recognition. ***A***, Schematic timeline of the experimental procedure. PBS or IBA was injected into the SuM at P35, and the three-chamber test (TCT) was conducted at P49. Brains were fixed on the day following the TCT. ***B***, Total interaction time (S1 + object) of the PBS- or IBA-injected mice. ***C***, The interaction time with S1 or the object for PBS- and IBA-injected mice. ***D***, Difference in interaction time with S1 versus the object (S1 − object) in PBS- and IBA-injected mice. See Extended Data Fig. 3-1 for the sociability discrimination index analysis. ***E***, Total interaction time (S2 + S1) of the test mouse, compared between PBS- and IBA-injected mice. ***F***, The interaction time with S1 or S2 for PBS- and IBA-injected mice. ***G***, Difference in the interaction time with S1 versus S2 (S2 − S1) between PBS- and IBA-injected mice. See Extended Data Figure 3-2 for the social novelty discrimination index analysis. Filled and unfilled circles in ***B***, ***D***, ***E***, and ***G*** represent data from individual mice. Filled blue and red circles in ***C*** and ***F*** represent data from individual mice (blue, S1; red, object in ***C***; and blue, S1; red, S2 in ***F***), and connecting lines represent the same mouse. ***H–M***, Images of the SuM in the medial position (SuMm) at A/P: −2.80 mm from bregma. Dotted lines outline the principal mammillary tract. Scale bar in ***H*** is 100 μm. ***H–J***, The SuMm region of PBS-injected mice showing Hoechst (***H***), NeuN^+^ cells detected by using immunohistochemistry (***I***), and merged Hoechst^+^/NeuN^+^ cells (***J***). ***K–M***, The SuMm region of IBA-injected mice showing Hoechst (***K***), NeuN^+^ cells (***L***), and merged Hoechst^+^/NeuN^+^ cells (***M***). ***N***, The number of NeuN^+^ cells in the SuM of PBS- or IBA-injected mice. Filled circles represent data from individual mice. All values are represented as mean ± SEM for ***B***, ***D***, ***E***, ***G***, and ***N***. *p < 0.05, **p < 0.01, ****p < 0.0001, ns, not significant. See Extended Data Figure 1-2 for detailed statistical information.

To test whether SuM function during puberty is necessary for adult social novelty recognition, we performed ibotenic acid (IBA) lesions of the medial SuM at P35 and evaluated social behavior at P49 (Fig. 3*A*). Sociability remained intact: total interaction time and preference for S1 over the object did not differ between PBS- and IBA-injected mice (Fig. 3*B–D*; Extended Data Fig. 3-1). In contrast, SuM lesions disrupted social novelty recognition. PBS-injected mice showed clear preference for S2 over S1, whereas IBA-injected mice did not (Fig. 3*E*,*F*). Although the S2–S1 difference did not reach statistical significance between groups, PBS mice showed a substantially longer mean difference (79.52 ± 22.01 s) compared with IBA mice (0.676 ± 35.94 s; Fig. 3*G*, Extended Data Fig. 3-2). NeuN staining confirmed a significant loss of SuM neurons in IBA-injected mice (661.2 ± 146.6 cells/mm^2^) relative to PBS controls (1,692 ± 164.4 cells/mm^2^), indicating successful lesion induction in the medial SuM (Fig. 3*H–N*). Together, these findings show that normal SuM development during puberty is essential for establishing adult social novelty recognition.

10.1523/ENEURO.0440-25.2026.f3-1 Figure 3-1 Discrimination index for the sociability phase for PBS- and IBA-injected mice. No significant difference of the preference toward S1 between PBS- and IBA-injected mice (*n* = 10 mice per group). ns., not significant. See Figure 1-2 for detailed statistical information. Download Figure 3-1, TIF file.

10.1523/ENEURO.0440-25.2026.f3-2 Figure 3-2 Discrimination index for the social novelty phase for PBS- and IBA-injected mice. No significant difference of the preference toward S2 between PBS- and IBA-injected mice (*n* = 10 mice per group). ns., not significant. See Figure 1-2 for detailed statistical information. Download Figure 3-2, TIF file.

### Impaired social novelty recognition in MS and *Caps2^−/−^* mice

**Figure 4.**
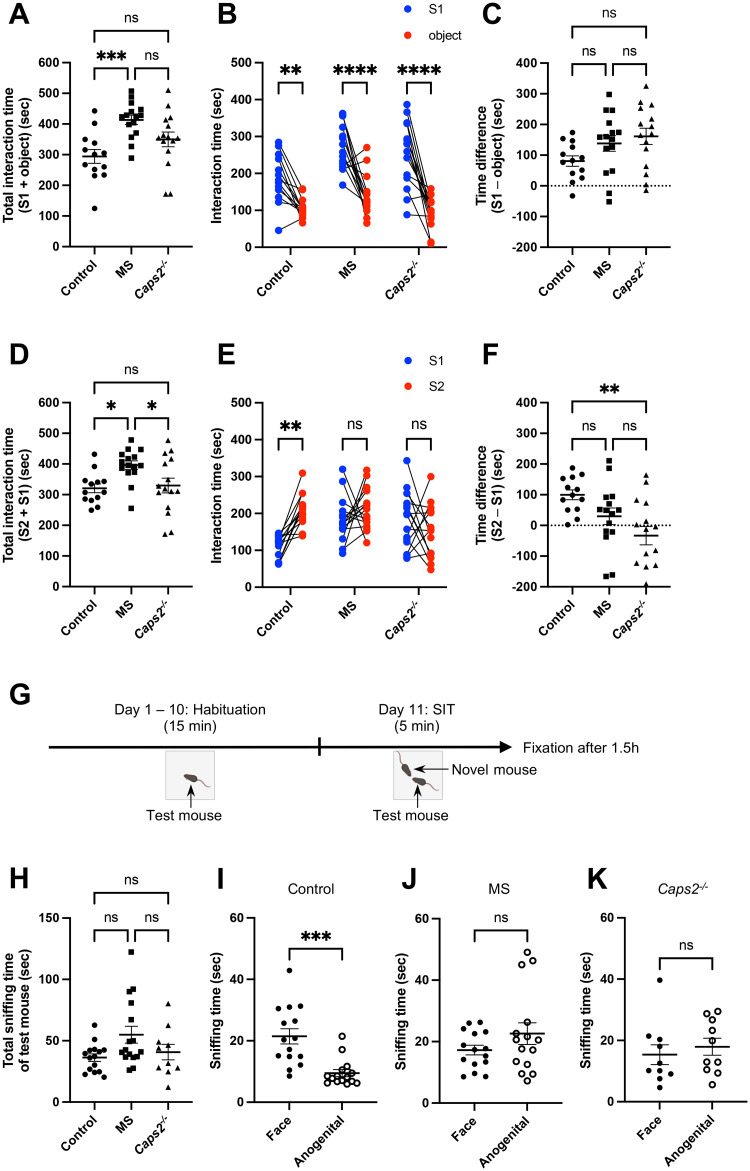
Social novelty recognition is impaired in MS and *Caps2^−/−^* mice. ***A***, Total exploration time for the stimulus mouse (S1) or the object by control, MS, and *Caps2^−/−^* mice. ***B***, Within-group comparison of interaction time with S1 or the object. ***C***, Difference in interaction time between S1 and the object among groups. See Extended Data Figure 4-1 for the sociability discrimination index analysis. ***D***, Total time interacting with the familiar (S1) and novel (S2) mouse (S2 + S1). ***E***, Within-group comparison of interaction time with S1 and S2. ***F***, Difference in interaction time between S1 and S2 among groups. See Extended Data Figure 4-2 for the social novelty discrimination index analysis. ***G***, Schematic diagram of the direct social interaction test (DSIT) and timeline of the experimental procedure. After a 10 d habituation period to the field, the test mouse was exposed to a novel mouse within the same field for 5 min on Day 11. Brains were collected and fixed 1.5 h after the interaction. ***H***, Total sniffing duration (face, body and anogenital) of the test mouse toward the novel mouse by control, MS, and *Caps2^−/−^* mice. ***I–K***, Duration of sniffing behavior exhibited by control (***I***), MS (***J***), and *Caps2^−/−^* (***K***) mice toward the face or anogenital region of the novel mouse. In ***A***, ***C***, ***D***, ***F***, and ***H***, filled circles, squares, and triangles represent individual mice. In ***B*** and ***E***, filled blue and red circles represent interaction time with S1 and the object/S2, respectively, with connecting lines indicating the same mouse. In ***I***, ***J***, and ***K***, filled and unfilled circles represent individual mice. All values are represented as mean ± SEM for ***A***, ***C***, ***D***, ***F***, ***H–K***. *p < 0.05, **p < 0.01, ***p < 0.001, ****p < 0.0001, ns, not significant. See Extended Data Figure 1-2 for detailed statistical information.

To compare SuM-related behavioral phenotypes across models, we assessed sociability and social novelty recognition in MS and *Caps2^−/−^* mice at P56. Sociability was preserved in both models, as all groups preferred S1 over the object (Fig. 4*A–C*). Total interaction time was increased in MS mice compared with controls, whereas no significant difference was observed between control and *Caps2^−/−^* mice (control: 80.7 ± 16.85 s; MS: 138.4 ± 25.56 s; *Caps2^−/−^*: 161.3 ± 26.43 s; Fig. 4*A*). However, preference for S1 over the object did not differ between groups (Fig. 4*A–C*; Extended Data Fig. 4-1). In contrast, both MS and *Caps2^−/−^* mice exhibited marked deficits in social novelty recognition (Fig. 4*D–F*). Unlike control mice, neither group showed preference for S2 over S1 (Fig. 4*D*,*E*). The S2–S1 difference score was significantly lower in *Caps2^−/−^* mice compared with controls, and MS mice showed no significant preference (control: 99.61 ± 16.04 s; MS: 29.61 ± 26.99 s; *Caps2^−/−^*: −33.14 ± 29.94 s; Fig. 4*F*, Extended Data Fig. 4-2). These results suggest that impairment in social novelty recognition may be more severe in *Caps2^−/−^* mice than in MS mice.

10.1523/ENEURO.0440-25.2026.f4-1 Figure 4-1 Discrimination index for the sociability phase for control, MS and *Caps2^-/-^* mice. No significant difference of the preference toward S1 between control, MS, and *Caps2^-/-^* mice (*n* = 13 mice for control, 15 mice for MS, 15 mice for *Caps2^-/-^*). ns., not significant. See Figure 1-2 for detailed statistical information. Download Figure 4-1, TIF file.

10.1523/ENEURO.0440-25.2026.f4-2 Figure 4-2 Discrimination index for the social novelty phase for control, MS and *Caps2^-/-^* mice. Control mice showed significantly stronger preference toward S2 than *Caps2^-/-^* mice (*n* = 13 for control, *n* = 15 for *Caps2^-/-^*), whereas control and MS mice showed a trend but did not reach significance (*n* = 15 for MS). No significant difference of the preference toward S2 between MS and *Caps2^-/-^* mice. *p < 0.05, ns., not significant. See Figure 1-2 for detailed statistical information. Download Figure 4-2, TIF file.

Next, direct social interactions between the test mouse and a novel mouse were compared among the three groups at P67 (Fig. 4*G*). Total sniffing duration did not differ across groups (Fig. 4*H*). However, control mice exhibited a clear preference for face-to-face over anogenital sniffing (face: 21.47 ± 2.479 s; anogenital: 9.478 ± 1.13 s; Fig. 4*I*). Both MS and *Caps2^−/−^* mice lacked this preference and showed similar durations for both types of contact (Fig. 4*J*,*K*). These results indicate that although general sociability is intact, MS and *Caps2^−/−^* mice both exhibit specific deficits in recognizing and preferentially investigating unfamiliar conspecifics.

### Reduced c-Fos activation in the SuM following social novelty exposure in MS and *Caps2^−/−^* mice

**Figure 5.**
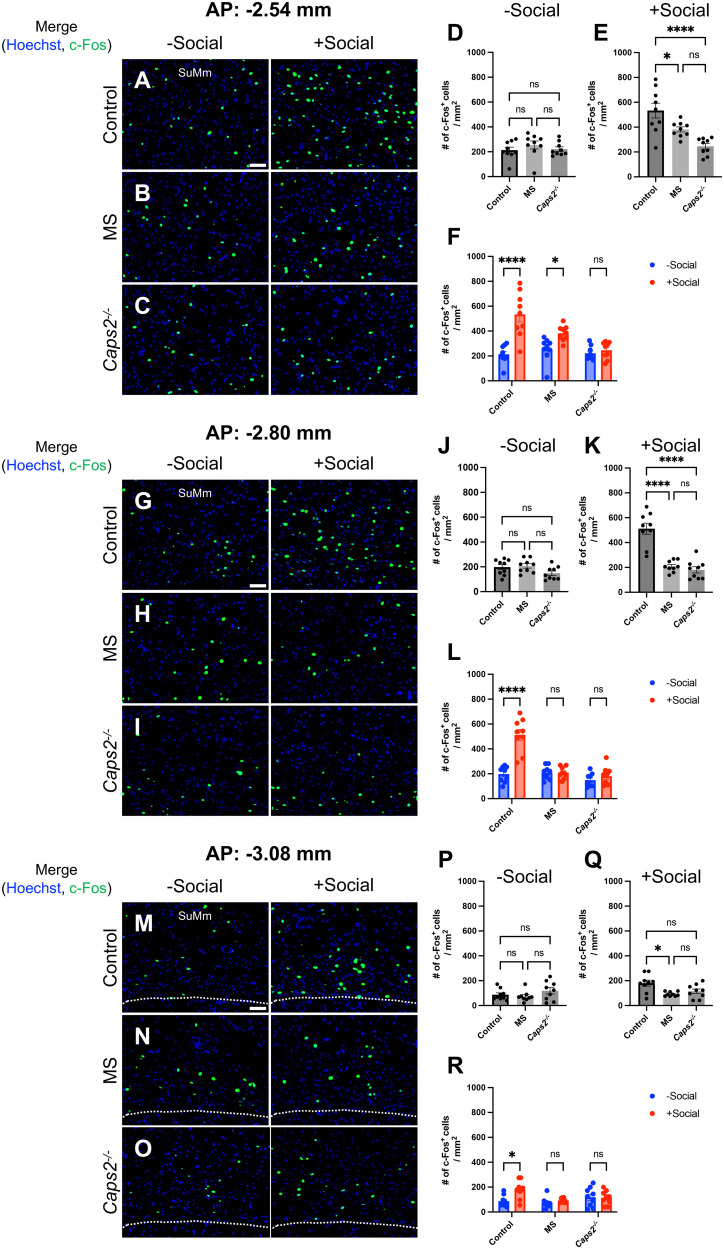
Social novelty-dependent c-Fos induction in the medial SuM of control, MS, and *Caps2^−/−^* mice across three anterior–posterior levels. c-Fos-positive cells were quantified in the medial SuM (mSuM) at three anterior–posterior levels (A/P −2.54 mm, −2.80 mm, and −3.08 mm; see Extended Data Figure 5-1 for anatomical locations of the quantified regions). ***A–C***, Representative images of c-Fos expression in the SuMm at A/P −2.54 mm from bregma in control (***A***), MS (***B***), and *Caps2^−/−^* (***C***) mice under the no-social (−social; left) and social novelty exposure (+social; right) conditions. ***D*–*F***, Quantification of c-Fos^+^ cell density in the SuMm at A/P −2.54 mm. ***D***, −social condition; ***E***, +social condition; ***F***, Comparison between the −social and +social conditions. ***G–I***, Representative images of c-Fos expression in the SuMm at A/P −2.80 mm from bregma in control (***G***), MS (***H***), and *Caps2^−/−^* (***I***) mice under the −social (left) and +social (right) conditions. ***J–L***, Quantification of c-Fos^+^ cell density in the SuMm at A/P −2.80 mm. ***J***, −social condition; ***K***, +social condition; ***L***, Comparison between the −social and +social conditions. ***M*–*O***, Representative images of c-Fos expression in the SuMm at A/P −3.08 mm from bregma in control (***M***), MS (***N***), and *Caps2^−/−^* (***O***) mice under the −social (left) and +social (right) conditions. ***P*–*R***, Quantification of c-Fos^+^ cell density in the SuMm at A/P −3.08 mm. ***P***, −social condition; ***Q***, +social condition; ***R***, comparison between the −social and +social conditions. Filled circles represent individual section values. Data are presented as mean ± SEM in ***D*–*F***, ***J*–*L***, and ***P*–*R***. Scale bar, 50 μm for all images. *p < 0.05, ****p < 0.0001; ns, not significant. See Extended Data Figure 1-2 for detailed statistical information.

To determine whether neuronal activation in the SuM in response to social novelty is altered in these models, we quantified c-Fos-positive cells in the SuM at three anterior–posterior levels (A/P −2.54 mm, −2.80 mm, and −3.08 mm; Extended Data Fig. 5-1) in P67 mice. In control mice, robust induction of c-Fos-positive cells in response to exposure to a novel mouse was observed at A/P −2.54 mm (−social: 212.7 ± 24.52 cells/mm^2^, +social: 533.4 ± 59.77 cells/mm^2^; Fig. 5*A*,*D*,*E*,*F*) and A/P −2.80 mm (−social: 198.2 ± 20.97 cells/mm^2^, +social: 511.9 ± 44.47 cells/mm^2^; Fig. 5*G*,*J*,*K*,*L*). c-Fos-positive cell induction was also observed at A/P −3.08 mm, although both baseline and socially induced c-Fos-positive cell densities were approximately half of those observed at A/P −2.54 mm and A/P −2.80 mm (−social: 87.24 ± 14.79 cells/mm^2^, +social: 180.6 ± 24.04 cells/mm^2^; Fig. 5*M*,*P*,*Q*,*R*). We also examined c-Fos induction in the lateral SuM (left lateral SuM: SuM_LL_, right lateral SuM: SuM_RL_ in Extended Data Figs. 8-1–8-3) and found that the induced cells were detected bilaterally at all three anterior–posterior levels, although the number was comparable to that observed in the medial SuM at A/P −3.08 mm (Fig. 8*Q*,*R*; Extended Data Figs. 8-1*I–L*, 8-2*I–L*, 8-3*I–L*).

10.1523/ENEURO.0440-25.2026.f5-1 Figure 5-1 Schematic diagrams of mouse coronal sections showing the SuM at A/P –2.54 mm, –2.80 mm, and –3.08 mm from bregma. The three boxed regions within the SuM indicate the left lateral SuM (SuM_LL_), medial SuM (SuMm), and right lateral SuM (SuM_RL_) subregions, where c-Fos⁺ and GPR54⁺ cells were quantified at P67. Download Figure 5-1, TIF file.

Social novelty-induced c-Fos activation was absent in *Caps2^−/−^* mice at all three anterior–posterior levels (A/P −2.54 mm: −social: 221.2 ± 18.17 cells/mm^2^, +social: 245.6 ± 22.7 cells/mm^2^, A/P −2.80 mm: −social: 148.5 ± 17.64 cells/mm^2^, +social: 180.6 ± 25.04 cells/mm^2^, A/P −3.08 mm: −social: 118.6 ± 25.35 cells/mm^2^, +social: 114.8 ± 18.33 cells/mm^2^; Fig. 5*C–F*,*I–L*,*O–R*). In contrast, a slight increase in c-Fos-positive cells was detected only in the anterior SuM at A/P −2.54 mm in MS mice following social novelty exposure (−social: 254.8 ± 31.87 cells/mm^2^, +social: 381.8 ± 20.21 cells/mm^2^; Fig. 5*B*,*D*,*E*,*F*). Social novelty-induced c-Fos activation disappeared in the SuM at A/P −2.80 mm and −3.08 mm in MS mice (A/P −2.80 mm: −social: 207.4 ± 18.17 cells/mm^2^, +social: 208.9 ± 15.27 cells/mm^2^, A/P −3.08 mm: −social: 73.46 ± 13.92 cells/mm^2^, +social: 92.59 ± 5.865 cells/mm^2^; Fig. 5*H*,*J*,*K*,*L*,*N*,*P*,*Q*,*R*).

In the lateral SuM, neither MS nor *Caps2^−/−^* mice exhibited detectable induction of c-Fos-positive cells at any anterior–posterior level following social novelty exposure (Extended Data Figs. 8-1*I–L,Q,R*, 8-2*I–L,Q,R*, 8-3*I–L,Q,R*). These results indicate that social novelty exposure robustly induced c-Fos activation predominantly in the anterior medial SuM (A/P −2.54 mm and −2.80 mm) in control mice. In contrast, this activation was largely absent in MS mice, except for a modest increase in the anterior medial SuM, and was undetectable in *Caps2^−/−^* mice.

### Reduced c-Fos activation in hippocampal CA2 following social novelty exposure in MS and *Caps2^−/−^* mice

**Figure 6.**
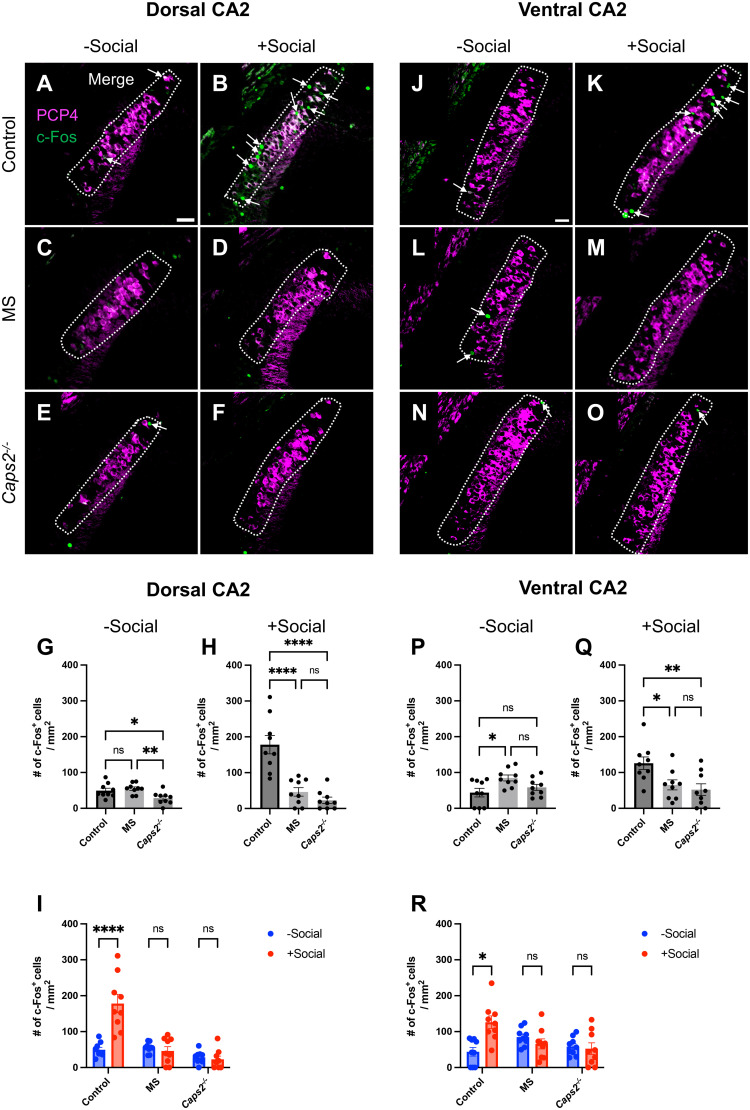
Reduced social novelty-dependent c-Fos induction in the dorsal and ventral CA2 of MS and *Caps2^−/−^* mice. ***A*–*F***, Representative images of merged PCP4 and c-Fos immunostaining in the dorsal CA2 at A/P −1.94 mm from bregma in control (***A***, ***B***), MS (***C***, ***D***), and *Caps2^−/−^* (***E***, ***F***) mice under the no-social (−social; left) and social novelty exposure (+social; right) conditions. ***G*–*I***, Quantification of c-Fos^+^ cell density in the dorsal CA2. ***G***, −social condition; ***H***, +social condition; ***I***, comparison between the −social and +social conditions. ***J*–*O***, Representative images of double immunostaining for PCP4 and c-Fos in the ventral CA2 at A/P −2.54 mm from bregma in control (***J***, ***K***), MS (***L***, ***M***), and *Caps2^−/−^* (***N***, ***O***) mice under the −social (left) and +social (right) conditions. White dotted lines in ***A*–*F*** and ***J*–*O*** outline the CA2 region identified by PCP4 expression. White arrows in ***A–O*** indicate c-Fos^+^ cells located within the PCP4-defined CA2 region. ***P*–*R***, Quantification of c-Fos^+^ cell density in the ventral CA2. ***P***, −social condition; ***Q***, +social condition; ***R***, comparison between the −social and +social conditions. Filled circles represent individual section values. Data are presented as mean ± SEM in ***G*–*I*** and ***P*–*R***. Scale bar, 50 μm for all images. *p < 0.05, **p < 0.01, ****p < 0.0001; ns, not significant. See Extended Data Figure 1-2 for detailed statistical information.

Next, we examined c-Fos induction in the hippocampal CA2 in response to social novelty exposure. Previous studies have demonstrated that CA2 is critically involved in social novelty recognition (Hitti and Siegelbaum, 2014; Chen et al., 2020). We examined c-Fos-positive cell counts in the dorsal and ventral CA2 of P67 mice exposed to social novelty versus unexposed controls. Dorsal and ventral CA2 regions were identified by PCP4 protein expression using immunohistochemistry, and the numbers of c-Fos-positive cells within these regions were compared. Consistent with previous studies, control mice exhibited robust induction of c-Fos-positive cells in both dorsal (−social: 49.65 ± 6.601 cells/mm^2^, +social: 178.2 ± 25.55 cells/mm^2^; Fig. 6*A*,*B*,*G*,*H*,*I*) and ventral CA2 (−social: 43.81 ± 12.01 cells/mm^2^, +social: 125.9 ± 17.52 cells/mm^2^; Fig. 6*J*,*K*,*P*,*Q*,*R*) following the social novelty exposure. In contrast, robust induction of c-Fos-positive cells in dorsal and ventral CA2 in response to social novelty exposure was absent both in MS and *Caps2^−/−^* mice (dorsal CA2 in MS: −social: 55.03 ± 4.801 cells/mm^2^, +social: 46.29 ± 11.95 cells/mm^2^, ventral CA2 in MS: −social: 84.71 ± 8.338 cells/mm^2^, +social: 65.51 ± 13.86 cells/mm^2^, dorsal CA2 in *Caps2^−/−^*: −social: 28.48 ± 5.696 cells/mm^2^, +social: 22.89 ± 8.768 cells/mm^2^, ventral CA2 in *Caps2^−/−^*: −social: 58.53 ± 8.423 cells/mm^2^, +social: 52.23 ± 16.11 cells/mm^2^; Fig. 6*C–I*,*L–R*). Under the no-exposure condition, the number of c-Fos-positive cells in dorsal CA2 was comparable between MS and control mice (MS: 55.03 ± 4.801 cells/mm^2^, control: 49.65 ± 6.601 cells/mm^2^), whereas *Caps2^−/−^* mice showed slightly fewer c-Fos-positive cells than both control and MS mice (*Caps2^−/−^* 28.48 ± 5.696 cells/mm^2^; Fig. 6*G*). The numbers of c-Fos-positive cells under the no-exposure condition in both MS and *Caps2^−/−^* mice were slightly higher than that of controls in the ventral CA2 (Fig. 6*P*). However, neither MS nor *Caps2^−/−^* mice showed c-Fos induction in response to social novelty exposure in either dorsal or ventral CA2 (Fig. 6*C–I*,*L–R*).

### Differential c-Fos induction in the DG following social novelty exposure in MS and *Caps2^−/−^* mice

**Figure 7.**
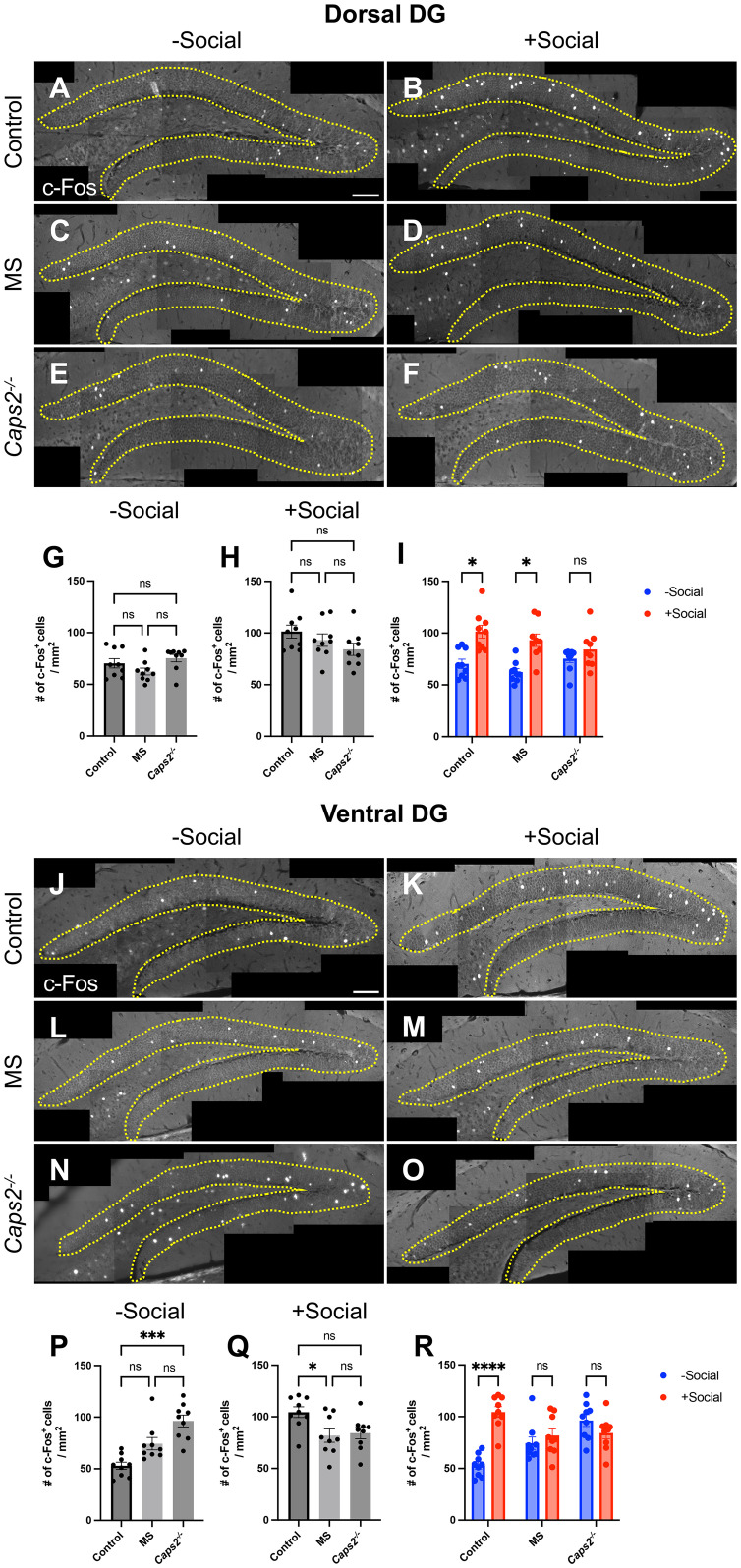
Differential social novelty-dependent c-Fos induction along the dorsoventral axis of the DG. ***A*–*F***, Representative images of c-Fos immunostaining in the dorsal DG at A/P −1.94 mm from bregma in control (***A***, ***B***), MS (***C***, ***D***), and *Caps2^−/−^* (***E***, ***F***) mice under the no-social (−social; left) and social novelty exposure (+social; right) conditions. ***G*–*I***, Quantification of c-Fos^+^ cell density in the dorsal DG. ***G***, −social condition; ***H***, +social condition; ***I***, comparison between the −social and +social conditions. ***J*–*O***, Representative images of c-Fos immunostaining in the ventral DG at A/P −2.54 mm from bregma in control (***J***, ***K***), MS (***L***, ***M***), and *Caps2^−/−^* (***N***, ***O***) mice under the −social (left) and +social (right) conditions. Yellow dotted lines in ***A*–*F*** and ***J*–*O*** outline the DG. ***P*–*R***, Quantification of c-Fos^+^ cell density in the ventral DG. ***P***, −social condition; ***Q***, +social condition; ***R***, comparison between the −social and +social conditions. Filled circles represent individual section values. Data are presented as mean ± SEM in ***G*–*I*** and ***P*–*R***. Scale bar, 100 μm for all images. *p < 0.05, ***p < 0.001, ****p < 0.0001; ns, not significant. See Extended Data Figure 1-2 for detailed statistical information.

Next, we examined c-Fos induction in the DG in response to social novelty exposure. In control mice, social novelty exposure induced robust c-Fos activation to a similar extent in both the dorsal (−social: 70.32 ± 4.532 cells/mm^2^, +social: 101.4 ± 6.286 cells/mm^2^; Fig. 7*A*,*B*,*G–I*) and ventral DG (−social: 52.95 ± 3.549 cells/mm^2^, +social: 104.5 ± 5.229 cells/mm^2^; Fig. 7*J*,*K*,*P–R*). The number of c-Fos-positive cells under the no-exposure condition was comparable among control (70.32 ± 4.532 cells/mm^2^), MS (62.4 ± 3.334 cells/mm^2^), and *Caps2^−/−^* mice (75.34 ± 3.629 cells/mm^2^) in the dorsal DG (Fig. 7*C*,*E*,*G*), whereas *Caps2^−/−^* mice (96.36 ± 5.743 cells/mm^2^) exhibited markedly elevated baseline c-Fos expression relative to control (52.95 ± 3.549 cells/mm^2^) and MS mice (74.3 ± 5.972 cells/mm^2^) in the ventral DG (Fig. 7*L*,*N*,*P*). Following social novelty exposure, MS mice exhibited a clear increase in dorsal DG c-Fos-positive cells comparable to that observed in control mice (−social: 62.4 ± 3.334 cells/mm^2^, +social: 93.16 ± 6.046 cells/mm^2^; Fig. 7*C*,*D*,*G–I*), whereas *Caps2^−/−^* mice showed no detectable induction in either the dorsal or ventral DG (dorsal: −social: 75.34 ± 3.629 cells/mm^2^, +social: 84.21 ± 5.889 cells/mm^2^, ventral: −social: 96.36 ± 5.743 cells/mm^2^, +social: 84.21 ± 5.527 cells/mm^2^; Fig. 7*E*,*F*,*G–I*,*N*,*O*,*P–R*). These results indicate that social novelty exposure-dependent activation in the DG is differentially affected across models and along the dorsal–ventral axis. Whereas dorsal DG activation was partially preserved in MS mice, novelty-induced activation was completely absent in *Caps2^−/−^* mice in both dorsal and ventral DG regions.

### GPR54 expression in the SuM shows divergent alterations in MS and *Caps2^−/−^* mice

**Figure 8.**
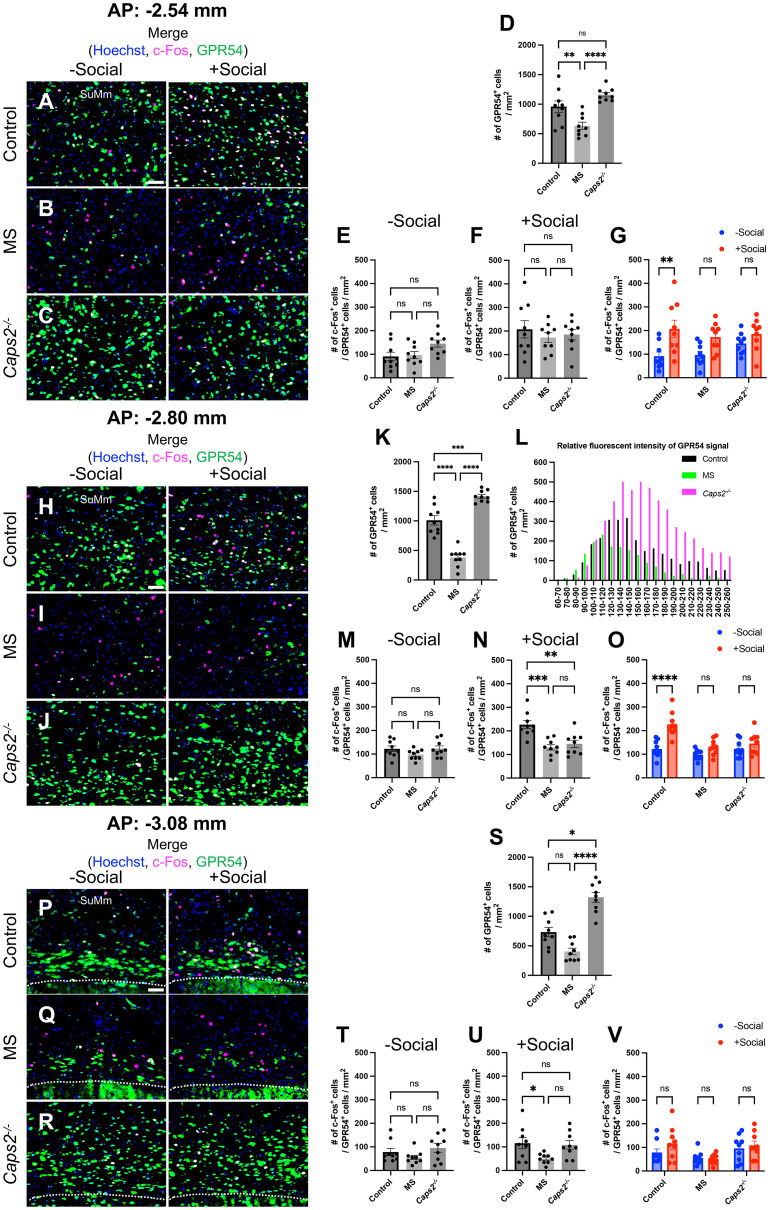
Relationship between c-Fos and GPR54 expression in the medial SuM of control, MS, and mice across three anterior–posterior levels. ***A*–*C***, Representative images of merged c-Fos and GPR54 immunostaining in the medial SuM (SuMm) at A/P −2.54 mm from bregma in control (***A***), MS (***B***), and *Caps2^−/−^* (***C***) mice under the no-social (−social; left) and social novelty exposure (+social; right) conditions. ***D***, Quantification of GPR54^+^ cell density in the SuMm at A/P −2.54 mm. ***E*–*G***, Quantification of c-Fos^+^/GPR54^+^ double-positive cell density in the SuMm at A/P −2.54 mm. ***E***, −social condition; ***F***, +social condition; ***G***, comparison between the −social and +social conditions. See Extended Data Figure 8-1 for corresponding analyses in the lateral SuM at A/P −2.54 mm from bregma. ***H*–*J***, Representative images of merged c-Fos and GPR54 immunostaining in the SuMm at A/P −2.80 mm from bregma in control (***H***), MS (***I***), and *Caps2^−/−^* (***J***) mice under the −social (left) and +social (right) conditions. ***K***, Quantification of GPR54^+^ cell density in the SuMm at A/P −2.80 mm. ***L***, Distribution of relative GPR54 fluorescence intensity in the SuMm. Black, green, and magenta bars indicate control (1,163 cells, 3 sections per mouse), MS (690 cells, 3 sections per mouse), and *Caps2^−/−^* mice (2,178 cells, 3 sections per mouse), respectively. Statistical Comparisons were not performed for these intensity distributions. ***M*–*O***, Quantification of c-Fos^+^/GPR54^+^ double-positive cell density in the SuMm at A/P −2.80 mm. ***M***, −social condition; ***N***, +social condition; ***O***, comparison between the −social and +social conditions. See Extended Data Figure 8-2 for corresponding analyses in the lateral SuM at A/P −2.80 mm from bregma. ***P*–*R***, Representative images of merged c-Fos and GPR54 immunostaining in the SuMm at A/P −3.08 mm from bregma in control (***P***), MS (***Q***), and *Caps2^−/−^* (***R***) mice under the −social (left) and +social (right) conditions. White dotted lines in ***P*–*R*** indicate the border between the SuMm and medial mammillary nucleus (MM). ***S***, Quantification of GPR54^+^ cell density in the SuMm at A/P −3.08 mm. ***T*–*V***, Quantification of c-Fos^+^/GPR54^+^ double-positive cell density in the SuMm at A/P −3.08 mm. ***T***, −social condition; ***U***, +social condition; ***V***, comparison between the −social and +social conditions. See Extended Data Figure 8-3 for corresponding analyses in the lateral SuM at A/P −3.08 mm from bregma. Filled circles represent individual section values. Data are presented as mean ± SEM in ***D*–*G***, ***K*–*O***, and ***S*–*V***. Scale bar, 50 μm for all images. *p < 0.05, **p < 0.01, ***p < 0.001, ****p < 0.0001; ns, not significant. See Extended Data Figure 1-2 for detailed statistical information.

Given the developmental increase in SuM GPR54 expression and its potential role in SuM excitability, we compared GPR54 expression in the medial SuM across three groups at A/P −2.54 mm, −2.80 mm, and −3.08 mm. Interestingly, GPR54-expressing cell density was consistently reduced across all three anterior–posterior levels of the SuM in MS mice (A/P −2.54 mm: 634.4 ± 60.89 cells/mm^2^, A/P −2.80 mm: 380.3 ± 51.32 cells/mm^2^; A/P −3.08 mm: 404.8 ± 56.11 cells/mm^2^; Fig. 8*B*,*D*,*I*,*K*,*Q*,*S*), reaching approximately half of the control level (A/P −2.54 mm: 959.6 ± 96.86 cells/mm^2^, A/P −2.80 mm: 1,010 ± 79.3 cells/mm^2^; A/P −3.08 mm: 733.1 ± 79.58 cells/mm^2^; Fig. 8*A*,*D*,*H*,*K*,*P*,*S*). In striking contrast, *Caps2^−/−^* mice exhibited a marked increase in GPR54-expressing cell density across all three levels, reaching approximately twice the control level (A/P −2.54 mm: 1,161 ± 37.53 cells/mm^2^, A/P −2.80 mm: 1,417 ± 32.52 cells/mm^2^; A/P −3.08 mm: 1,322 ± 84.42 cells/mm^2^; Fig. 8*C*,*D*,*J*,*K*,*R*,*S*). Relative fluorescence intensity measurements at A/P −2.80 mm of the SuM yielded similar results, with weaker GPR54 signals in MS mice and stronger signals in *Caps2^−/−^* mice (Fig. 8*L*).

10.1523/ENEURO.0440-25.2026.f8-1 Figure 8-1 *A–F*, Representative images of merged Hoechst/c-Fos/GPR54 immunofluorescence signals in the left lateral SuM (SuM_LL_; left images) and right lateral SuM (SuM_RL_; right images) at A/P –2.54 mm from bregma under the –social (*A–C*) and +social (*D–F*) conditions in control (*A, D*), MS (*B, E*), and *Caps2^-/-^* (*C*, *F*) mice. White dotted lines indicate the borders between the SuM and medial mammillary nucleus (MM). Scale bar = 50 μm. *G, H,* GPR54⁺ cell density in the SuM_LL_ (*G*) and SuM_RL_ (*H*). *I, J,* c-Fos⁺ cell density under the –social condition in the SuM_LL_ (*I*) and SuM_RL_ (*J*). *K, L,* c-Fos⁺ cell density under the +social condition in the SuM_LL_ (*K*) and SuM_RL_ (*L*). *M, N*, c-Fos⁺/GPR54⁺ cell density under the –social condition in the SuM_LL_ (*M*) and SuM_RL_ (*N*). *O, P*, c-Fos⁺/GPR54⁺ cell density under the +social condition in the SuM_LL_ (*O*) and SuM_RL_ (*P*). *Q, R*, Comparison of c-Fos⁺ cell density between the –social and +social conditions in the SuM_LL_ (*Q*) and SuM_RL_ (*R*). *S, T*, Comparison of c-Fos⁺/GPR54⁺ cell density between the –social and +social conditions in the SuM_LL_ (*S*) and SuM_RL_ (*T*). n = 3 sections per mouse, 3 mice per group. Filled circles represent values from individual sections. Data are presented as mean ± SEM (*G–T*). *p < 0.05, **p < 0.01, ***p < 0.001, ****p < 0.0001; ns, not significant. See Figure 1-2 for detailed statistical information. Download Figure 8-1, TIF file.

10.1523/ENEURO.0440-25.2026.f8-2 Figure 8-2 *A–F*, Representative images of merged Hoechst/c-Fos/GPR54 immunofluorescence signals in the left lateral SuM (SuM_LL_; left images) and right lateral SuM (SuM_RL_; right images) at A/P –2.80 mm from bregma under the –social (*A–C*) and +social (*D–F*) conditions in control (*A, D*), MS (*B, E*), and *Caps2^-/-^* (*C*, *F*) mice. White dotted lines indicate the borders between the SuM and medial mammillary nucleus (MM). Scale bar = 50 μm. *G, H,* GPR54⁺ cell density in the SuM_LL_ (*G*) and SuM_RL_ (*H*). *I, J,* c-Fos⁺ cell density under the –social condition in the SuM_LL_ (*I*) and SuM_RL_ (*J*). *K, L,* c-Fos⁺ cell density under the +social condition in the SuM_LL_ (*K*) and SuM_RL_ (*L*). *M, N*, c-Fos⁺/GPR54⁺ cell density under the –social condition in the SuM_LL_ (*M*) and SuM_RL_ (*N*). *O, P*, c-Fos⁺/GPR54⁺ cell density under the +social condition in the SuM_LL_ (*O*) and SuM_RL_ (*P*). *Q, R*, Comparison of c-Fos⁺ cell density between the –social and +social conditions in the SuM_LL_ (*Q*) and SuM_RL_ (*R*). *S, T*, Comparison of c-Fos⁺/GPR54⁺ cell density between the –social and +social conditions in the SuM_LL_ (*S*) and SuM_RL_ (*T*). n = 3 sections per mouse, 3 mice per group. Filled circles represent values from individual sections. Data are presented as mean ± SEM (*G–T*). *p < 0.05, **p < 0.01, ***p < 0.001, ****p < 0.0001; ns, not significant. See Figure 1-2 for detailed statistical information. Download Figure 8-2, TIF file.

10.1523/ENEURO.0440-25.2026.f8-3 Figure 8-3 *A–F*, Representative images of merged Hoechst/c-Fos/GPR54 immunofluorescence signals in the left lateral SuM (SuM_LL_; left images) and right lateral SuM (SuM_RL_; right images) at A/P –3.08 mm from bregma under the –social (*A–C*) and +social (*D–F*) conditions in control (*A, D*), MS (*B, E*), and *Caps2^-/-^* (*C*, *F*) mice. White dotted lines indicate the borders between the SuM and medial mammillary nucleus (MM). Scale bar = 50 μm. *G, H,* GPR54⁺ cell density in the SuM_LL_ (*G*) and SuM_RL_ (*H*). *I, J,* c-Fos⁺ cell density under the –social condition in the SuM_LL_ (*I*) and SuM_RL_ (*J*). *K, L,* c-Fos⁺ cell density under the +social condition in the SuM_LL_ (*K*) and SuM_RL_ (*L*). *M, N*, c-Fos⁺/GPR54⁺ cell density under the –social condition in the SuM_LL_ (*M*) and SuM_RL_ (*N*). *O, P*, c-Fos⁺/GPR54⁺ cell density under the +social condition in the SuM_LL_ (*O*) and SuM_RL_ (*P*). *Q, R*, Comparison of c-Fos⁺ cell density between the –social and +social conditions in the SuM_LL_ (*Q*) and SuM_RL_ (*R*). *S, T*, Comparison of c-Fos⁺/GPR54⁺ cell density between the –social and +social conditions in the SuM_LL_ (*S*) and SuM_RL_ (*T*). n = 3 sections per mouse, 3 mice per group. Filled circles represent values from individual sections. Data are presented as mean ± SEM (*G–T*). *p < 0.05, **p < 0.01, ***p < 0.001, ****p < 0.0001; ns, not significant. See Figure 1-2 for detailed statistical information. Download Figure 8-3, TIF file.

We next examined social novelty-related activation of GPR54-expressing cells in the medial SuM. In control mice, social novelty exposure induced an approximately twofold increase in the number of c-Fos/GPR54-expressing cells in the anterior medial SuM (A/P −2.54 mm: −social: 91.06 ± 18.03 cells/mm^2^, +social: 207.4 ± 36.67 cells/mm^2^; and A/P −2.80 mm: −social: 122.4 ± 11.92 cells/mm^2^, +social: 226.5 ± 17.23 cells/mm^2^) compared with the no-exposure condition, whereas no such increase was observed in the posterior medial SuM (A/P −3.08 mm: −social: 78.05 ± 15.23 cells/mm^2^, +social: 115.5 ± 23.49 cells/mm^2^; Fig. 8*E–G*,*M–O*,*T–V*). In contrast, no increase in c-Fos/GPR54-expressing cells was observed after social novelty exposure in the medial SuM of both MS (A/P −2.54 mm: −social: 96.42 ± 15.44 cells/mm^2^, +social: 172.2 ± 20.4 cells/mm^2^, A/P −2.80 mm: −social: 97.95 ± 7.239 cells/mm^2^, +social: 130.1 ± 11.27 cells/mm^2^, and A/P −3.08 mm: −social: 55.1 ± 9.183 cells/mm^2^, +social: 50.51 ± 6.982 cells/mm^2^) and *Caps2^−/−^* mice (A/P −2.54 mm: −social: 145.4 ± 14.67 cells/mm^2^, +social: 185.2 ± 22.3 cells/mm^2^, A/P −2.80 mm: −social: 123.2 ± 11.73 cells/mm^2^, +social: 145.4 ± 15.42 cells/mm^2^, and A/P −3.08 mm: −social: 95.65 ± 18.45 cells/mm^2^, +social: 108.7 ± 18.14 cells/mm^2^; Fig. 8*E–G*,*M–O*,*T–V*). These results demonstrate that MS and *Caps2^−/−^* mice exhibit opposite patterns of GPR54 expression in the medial SuM, despite sharing impaired social novelty recognition and reduced novelty-dependent neuronal activation.

## Discussion

We investigated how the SuM responds to social novelty exposure in two neurodevelopmental disorder models with distinct etiologies: an early-life stress model induced by maternal separation (MS) and a neuropeptide secretion-deficient model using *Caps2^−/−^* mice. Despite their different developmental backgrounds, neither model exhibited social novelty-induced c-Fos expression in the SuM. Furthermore, although social novelty recognition was already established by P35, SuM lesions performed at P35 abolished this ability in adulthood. These findings suggest that intact SuM function during puberty is important for the establishment of adult social novelty recognition. They also raise the possibility that SuM dysfunction contributes to impaired social novelty recognition across distinct neurodevelopmental disorder models. In addition, the abnormal GPR54 expression observed in both models raises the possibility that altered KISS1–GPR54 signaling may be associated with impaired SuM activation and social novelty recognition.

### Potential relationship between SuM maturation and GPR54 signaling

GPR54 is a G-protein–coupled receptor for the neuropeptide KISS1. The KISS1–GPR54 signaling pathway is known to play an essential role in the onset of puberty by initiating reproductive hormone release through activation of hypothalamic GnRH neurons (Kotani et al., 2001; Ohtaki et al., 2001; Han et al., 2005). Beyond its classical role in reproductive regulation, GPR54-expressing neurons have also been identified in multiple nuclei associated with arousal, sensory processing, and limbic functions, suggesting that KISS1 signaling may contribute more broadly to neuromodulatory processes in the brain (Zhang et al., 2025). We found that both the number and expression levels of GPR54-expressing neurons in the SuM increased developmentally from P35 to P56. This increase coincided with the age-dependent enhancement of social investigation toward novel conspecifics (Figs. 1, 2). This developmental period overlaps with the increase in KISS1-expressing neurons reported in the anteroventral periventricular nucleus (AVPV) and the preoptic periventricular nucleus (PeN) between P25 and P61 (Clarkson and Herbison, 2006). These findings suggest that developmental increases in KISS1-expressing neurons, GPR54 expression in the SuM, and novelty-induced SuM activation appear to occur in parallel between P25 and P61. Because GPR54 activation engages intracellular pathways that enhance neuronal excitability and synaptic plasticity, KISS1–GPR54 signaling may contribute to SuM circuit maturation during puberty (Zhang et al., 2008; Szereszewski et al., 2010). Although *Gpr54* and *Kiss1* knock-out models have been extensively studied in reproductive physiology, their roles in nonreproductive social behavior remain largely unexplored. Our findings raise the possibility that GPR54 signaling may be associated with SuM-mediated social novelty processing. Interestingly, *Caps2^−/−^* mice exhibited an increased number of GPR54-positive cells, whereas MS mice showed reduced GPR54 expression levels (Fig. 8; Extended Data Fig. 8-1, 8-2, 8-3). These observations suggest that GPR54 expression itself may not directly reflect SuM neuronal excitability or socially induced activation. Instead, altered KISS1–GPR54 signaling may be associated with impaired SuM responses across the two models. Given the opposite GPR54 expression phenotypes observed in MS and *Caps2^−/−^* mice, future studies should clarify how altered KISS1 signaling contributes to the impaired novelty-dependent activation of SuM neurons in both models. Further studies are needed to determine whether neonatal chronic stress induced by MS or impaired neuropeptide secretion caused by CAPS2 deficiency directly or indirectly disrupts KISS1–GPR54 signaling.

### MS mice: reduced GPR54 expression and loss of SuM activation

MS mice exhibited markedly reduced numbers of GPR54-positive cells and reduced GPR54 expression levels in the SuM together with impaired c-Fos induction following social novelty exposure (Figs. 5, 8). Stress-dependent suppression of Kiss1 and Gpr54 mRNA expression has also been reported through corticotropin-releasing hormone (CRH) signaling in the adult brain. In the arcuate nucleus (ARC), stress input suppresses KISS1 expression through CRH signaling (Takumi et al., 2012; Ozawa, 2021). In addition, intracerebral administration of CRH suppresses not only *Kiss1* but also *Gpr54* expression in the adult medial preoptic area (mPOA; Kinsey-Jones et al., 2009). Chronic stress induced by MS has also been reported to increase CRH mRNA expression in the rat pups (Plotsky and Meaney, 1993). KISS1-expressing neurons begin developing at approximately P10, and their numbers increase until P61 in the AVPV, PeN, and ARC of the mouse hypothalamus (Clarkson and Herbison, 2006). Thus, elevated CRH signaling induced by MS may disrupt both *Kiss1* and *Gpr54* expression during postnatal brain development. Collectively, these mechanisms may contribute to the reduced GPR54 expression and impaired novelty-dependent SuM activation observed in MS mice. Future studies examining the number, morphology, and transcriptional regulation of KISS1-expressing neurons in MS mice will be important for clarifying relationships between stress, GPR54 expression, and SuM dysfunction.

### *Caps2^−/−^* mice: increased GPR54 expression despite loss of SuM activation

In contrast to MS mice, *Caps2^−/−^* mice exhibited markedly increased numbers of GPR54-positive cells and elevated GPR54 expression levels in the SuM yet failed to induce c-Fos expression in the SuM following social novelty exposure.

CAPS2 regulates dense-core vesicle trafficking and secretion of neuropeptides, including BDNF and neurotrophin-3 (NT-3), as well as dopamine release (Shinoda et al., 2011; Sadakata et al., 2012). Therefore, neuromodulatory signaling pathways including KISS1 signaling may also be disrupted in *Caps2^−/−^* mice. Most KISS1 neurons in the lateral septum project to and terminate within the SuM (Szentkirályi-Tóth et al., 2025). Because KISS1 signaling has been implicated in SuM excitability and maturation, increased GPR54 expression in *Caps2^−/−^* mice may reflect compensatory receptor upregulation in response to reduced ligand-dependent signaling, similar to that reported for neuropeptide Y receptors and opioid receptor-like 1 (ORL1) receptors in mutant mice lacking their respective ligand genes (Trivedi et al., 2001; Clarke et al., 2003). However, despite this apparent increase in GPR54, novelty-dependent SuM activation was not restored. This finding raises the possibility that KISS1-mediated input to SuM neurons may be impaired due to CAPS2-dependent deficits in vesicle trafficking or secretion. At present, aside from BDNF, NT-3, and dopamine, little direct evidence exists regarding altered neuropeptide or neurotransmitter release from axon terminals in *Caps2^−/−^* mutants, and the full range of neuropeptides regulated by CAPS2-dependent trafficking and secretion remains unknown (Sadakata et al., 2004; Shinoda et al., 2011; Iguchi et al., 2024). Determining whether KISS1 secretion is disrupted in *Caps2^−/−^* mice will therefore be an important direction for future research. Furthermore, testing whether exogenous administration of neuropeptides such as KISS1 can restore novelty-dependent SuM activation following social interaction may help clarify whether neuromodulatory signaling deficits contribute to impaired SuM responsiveness and social novelty recognition.

### Relationship with the hippocampus

In control mice, social novelty exposure induced robust c-Fos expression in the SuM together with strong c-Fos induction in the dorsal CA2 region of the hippocampus (Fig. 6). Recent studies have shown that SuM neurons projecting to CA2 preferentially respond to social novelty, whereas SuM neurons projecting to the DG encode contextual novelty (Chen et al., 2020). Thus, the social novelty exposure-dependent c-Fos induction observed in dorsal CA2 may be associated with activity of SuM neurons projecting to dorsal CA2. Interestingly, c-Fos induction was also observed in ventral CA2. Although dorsal CA2 has been implicated in social novelty recognition, ventral CA2 may also contribute to this process (Boyle et al., 2025). Importantly, social novelty-related c-Fos induction in both dorsal and ventral CA2 was absent in MS and *Caps2^−/−^* mice. These findings raise the possibility that SuM dysfunction in the two models may disrupt social novelty exposure-dependent neuronal activation in both dorsal and ventral CA2. However, because projections from the SuM to CA2 were not directly examined in this study, further investigation will be necessary to determine whether the observed ventral CA2 activation is SuM dependent.

In addition to the CA2, we also observed robust c-Fos induction in both the dorsal and ventral DG following social novelty exposure (Fig. 7). Although the relationship between these DG responses and SuM activity remains unclear, c-Fos induction in the ventral DG was absent in both MS and *Caps2^−/−^* mice. The absence of ventral DG activation in mice with impaired social novelty recognition raises the possibility that the ventral DG may also participate in processing social novelty-related information. Indeed, a subset of SuM neurons projecting to the hippocampus has been reported to respond to both social and contextual novelty (Chen et al., 2020).

### Divergent molecular pathology but convergent circuit dysfunction

Although MS and *Caps2^−/−^* mice exhibited opposite changes in GPR54 expression, both models displayed impaired social novelty recognition and failed to induce c-Fos expression in the SuM following social novelty exposure. These findings suggest that SuM dysfunction may contribute to impaired social novelty recognition shared across these two neurodevelopmental disorder models. Our results further suggest that SuM maturation may depend on neuromodulatory signaling mechanisms including the KISS1–GPR54 pathway during the transition from puberty to adulthood. Disruption of these signaling mechanisms by early-life stress or impaired neuropeptide secretion may impair social novelty exposure-dependent activation of SuM neurons in response to social novelty exposure, thereby impairing hippocampal social novelty processing. This framework may help explain social novelty deficits observed across distinct neurodevelopmental disorders, including ASD and stress-induced developmental abnormalities. Future work should investigate the projection targets and functional manipulation of GPR54-expressing neurons within the SuM in both models, characterize the activity dynamics of these neurons in response to kisspeptin signaling, and identify developmental periods during which SuM maturation is particularly vulnerable. Such studies may help identify candidate targets for restoring social recognition function in neurodevelopmental disorders.

### Limitation

Although the present study focused on the SuM, this region receives diverse neural inputs from multiple brain areas involved in processing various types of information. Therefore, we cannot exclude the possibility that abnormalities outside the SuM contribute to the impaired c-Fos induction observed in the SuM. In addition, although altered GPR54 expression was observed in the SuM, we did not examine GPR54 expression in other brain regions or directly test the functional relationship between Kiss1–GPR54 signaling and SuM neuronal activation. Therefore, the potential involvement of Kiss1–GPR54 signaling in the impaired c-Fos response remains to be elucidated. Future studies will be required to clarify the circuit- and molecular-level mechanisms underlying SuM dysfunction in these models.

In MS mice, early-life stress may broadly suppress gene expression and impair postnatal neural development, potentially affecting multiple neural circuits. However, it remains unclear which specific neural circuits are disrupted by MS. In *Caps2^−/−^*mice, although CAPS2 deficiency is known to impair axonal transport and dense-core vesicle-dependent neuropeptide secretion, the range of neuropeptides regulated in a CAPS2-dependent manner remains incompletely understood. To date, only a limited number of pathways, including oxytocin and BDNF signaling, have been characterized.

In addition, the present study primarily relied on c-Fos expression as an indirect marker of neuronal activation. Although c-Fos analysis is widely used to identify neurons activated in response to social stimuli, it does not directly demonstrate functional synaptic connectivity or causal circuit interactions between the SuM and hippocampal regions such as CA2 or DG. Furthermore, projections from the SuM to dorsal and ventral hippocampal subregions were not directly examined in this study. Therefore, whether the observed hippocampal activation patterns are directly mediated by circuits involving the SuM remains to be determined.

Another limitation is that the present study focused exclusively on male mice. We used only male mice for two main reasons. First, this approach allowed direct continuity and comparison with previous foundational studies of *Caps2^−/−^* mice, which primarily examined male social behaviors. Second, because the female estrous cycle is known to modulate social novelty recognition, restricting the analysis to males helped minimize potential variability when assessing baseline behavioral phenotypes in these models. Nevertheless, given the known sex differences in ASD prevalence and symptom presentation, examining sex-dependent differences in circuit-level, molecular, and functional abnormalities in these models will be an important direction for future research.

Future studies combining circuit tracing, in vivo activity recording, optogenetic or chemogenetic manipulation, and neuropeptide signaling analyses will be necessary to determine whether distinct neurodevelopmental perturbations converge onto common SuM-dependent mechanisms underlying social novelty recognition or instead diverge into model-specific pathways.

## Synthesis

Reviewing Editor: Katrina Choe, McMaster University Decisions are customarily a result of the Reviewing Editor and the peer reviewers coming together and discussing their recommendations until a consensus is reached. When revisions are invited, a fact-based synthesis statement explaining their decision and outlining what is needed to prepare a revision will be listed below. The following reviewer(s) agreed to reveal their identity: Satoshi Ikemoto.

This is a potentially interesting article showing differences in the supramammillary nucleus (SuM) and CA2 region of the hippocampus in mice subjected to maternal separation or are lacking the Caps2 gene. The authors show that both models show impaired social recognition, and exhibited fewer c-Fos+ cells in both the SuM and CA2 after novel social encounters compared to controls. Analysis of a signaling regulatory molecule GPR54 suggests alterations in the SuM of both models but in opposite directions. The reviewers found these results interesting and novel, and also commented positively on the clear writing of the manuscript. However, they also raised the following concerns listed below:

Major:

1. Provide a rationale for why only males were used. Provide additional details about the maternal separation paradigm - were the pups cross fostered, were only male pups subjected to maternal separation or was the entire litter subjected to the paradigm but only males tested after weaning?

2. More details should be provided about the c-fos experiment. It is not clear whether all or only some of the data were taken after social novelty exposure. What was the time point after social novelty exposure that the mice were perfused? Were home cage controls included? This information is needed because results are impossible to interpret as it stands.

3. The CA2 region is known to be molecularly distinguishable, which is typically the way that it is assessed and separated from CA1 and CA3 in histology studies. The researchers do not mention using a CA2 marker and instead indicate that they used an atlas to delineate the structure. It would be better to use a CA2 marker. Also, the images in Figure appear to be from ventral CA2, not dorsal CA2. Because dorsal CA2 is most closely linked to social novelty exposure, the analysis should assess this region. More details and better neuroanatomical localization should be provided.

4. The phrase "SuM-CA2 pathway" should be used more cautiously. As currently written, it implies that the present study directly examined this pathway, which it did not, and is therefore potentially misleading. While previous studies suggest that the SuM-CA2 pathway plays an important role in social memory, the present study assessed c-Fos expression in the SuM and CA2 independently. Consequently, it remains unclear whether c-Fos expression in SuM neurons led to c-Fos induction in CA2 neurons. The SuM projects to regions other than the CA2 and there are also reciprocal CA2-SuM projections. Accordingly, the statement on p. 14-"These findings demonstrate that deficits in social novelty recognition in both models are accompanied by a failure to activate the SuM-CA2 pathway"-should be tempered to more accurately reflect the data.

5. GPR54-positive cells were quantified across the entire SuM, whereas c-Fos was quantified within a restricted midline region (0.145 mm²; A/P −2.80 mm). Similarly, lesions were targeted to the midline SuM at approximately A/P −2.50 mm. Additional justification or clarification is needed to explain these differences in anatomical sampling and targeting. Particularly, do these choices have anything to do with selective projection of midline SuM neurons to the CA2? The GPR54 data are interesting but they are far from conclusive. It would be good to temper the discussion of these findings and to suggest how establishing causality could be achieved.

Minor issues:

• Cell counts should be expressed as /mm^2 or /um^2 instead of /0.145 mm^2 or /10^4 um^2

• p. 2: Although the study shows that maternal separation and Caps2 deletion reduce c-Fos expression following social interaction relative to controls, it does not demonstrate a complete absence of c-Fos induction. To support such a claim, control groups without social interaction would be required. I recommend rephrasing "no c-Fos induction."

• p. 6: Remove the "J" from "C57BL/6J," as this designation specifically indicates mice sourced from The Jackson Laboratory.

## Author Response

Manuscript ID: eN-NWR-0440-25 Manuscript Title: Reduced SuM Activation Accompanies Impaired Social Novelty Recognition in Mouse Models of Neurodevelopmental Disorders Date: 25 May 2026 Dear Dr. Choe and Reviewers, We appreciate you and the reviewers for their careful evaluation of our manuscript and for their constructive comments. We have addressed all concerns raised and revised the manuscript accordingly. We improved unclear technical descriptions throughout the manuscript and, in accordance with the reviewers' suggestions, performed additional experiments to more rigorously compare neuronal populations responsive to social novelty, the results of which have been incorporated into the revised version. We also made substantial efforts to ensure that our conclusions remain strictly grounded in the experimental findings without including excessive speculation. These revisions have greatly improved the accuracy and clarity with which the present study conveys its findings. We sincerely appreciate the reviewers' thoughtful and constructive comments.

Major Concerns Major 1:

1. Provide a rationale for why only males were used.

2. Provide additional details about the maternal separation paradigm - were the pups cross fostered, were only male pups subjected to maternal separation or was the entire litter subjected to the paradigm but only males tested after weaning? Response: 1-1 We apologize for the insufficient description of the methods. We used only male mice based on two main considerations: (1) Using male mice allows for direct continuity and comparison with previous foundational studies on Caps2-/- mice, which primarily evaluated male social behaviors; and (2) to clearly assess the baseline behavioral phenotypes in this model, it was necessary to exclude the influence of the female estrous cycle, which is known to strongly modulate social novelty recognition; therefore, female animals were not included in this study.

However, we acknowledge the reviewer's valid point regarding the importance of sex differences. The exclusion of female mice is a limitation of the present study. Given the known sex differences in ASD prevalence and symptom presentation, examining sex differences in circuit-level molecular and functional abnormalities in ASD models will be an important direction for future research. This limitation has been explicitly stated in the Limitation section of the revised manuscript (Limitation; p. 29, lines 3-12).

Response: 1-2 Regarding the detailed procedure of the maternal separation (MS) paradigm, we did not employ a cross-fostering paradigm. Only male pups were separated from the dam, whereas female pups remained in the home cage during the separation period. This partial-litter separation design was adopted based on previous studies aiming to minimize excessive disruption of maternal care caused by complete litter removal while still inducing early-life social stress in male pups. However, maternal behavior was not quantitatively assessed in the present study, and therefore potential effects of altered dam-pup interactions cannot be excluded. We have clarified these methodological details in the revised manuscript (Materials and methods; p. 6, line 21-p. 7, line 1).

Major 2:

1. More details should be provided about the c-fos experiment. It is not clear whether all or only some of the data were taken after social novelty exposure.

2. What was the time point after social novelty exposure that the mice were perfused? 3. Were home cage controls included? This information is needed because results are impossible to interpret as it stands.

Response 2-1:

We apologize for the lack of clarity in our initial manuscript. In the revised manuscript, we presented the results of all c-fos expression in the SuM, CA2 and DG both with and without social novelty exposure (Figure 5, 6, 7, 8, Extended data, Figure 8-1, 8-2, 8-3).

Response 2-2:

Regarding the time point after social novelty exposure, all mice for detecting c-fos in this study were perfused exactly 1.5 hours after the direct social interaction test. We described the time point in the new section entitled as "Social novelty exposure and c-Fos detection" in the Materials &methods to explain this procedure (Materials and Methods; p. 11, line 12-24).

Response 2-3:

Were home cage controls included?: Regarding the home cage control condition, only WT control data were included in the supplementary figure of the previous manuscript.

To more rigorously evaluate c-Fos induction associated with social novelty exposure across all three groups (Control, MS, and Caps2-/-), we newly added matched control groups without social novelty exposure. These control animals underwent the same experimental procedures as the social novelty exposure group. Specifically, all mice received 10 days of habituation to the test arena while group-housed from P57. Immediately following the habituation session on Day 10, mice were single-housed to minimize background c-Fos expression potentially associated with social interactions prior to testing. On Day 11, experimental mice were exposed to a novel mouse, whereas control mice were placed individually in the same arena for the identical duration without a stimulus mouse. This revised protocol allows clearer interpretation of c-Fos induction associated with social novelty exposure. These methodological details have been added to the new section entitled "Social novelty exposure and c-Fos detection" in the (Materials and Methods; p. 11, line 12-24).

Major 3:

1. The CA2 region is known to be molecularly distinguishable, which is typically the way that it is assessed and separated from CA1 and CA3 in histology studies. The researchers do not mention using a CA2 marker and instead indicate that they used an atlas to delineate the structure. It would be better to use a CA2 marker.

2. Also, the images in Figure appear to be from ventral CA2, not dorsal CA2. Because dorsal CA2 is most closely linked to social novelty exposure, the analysis should assess this region. More details and better neuroanatomical localization should be provided.

Response 3-1:

We fully agree with the reviewer that molecular identification of the CA2 region is important for accurate neuroanatomical delineation. In the revised manuscript, we used PCP4 immunostaining as a molecular marker to define both dorsal and ventral CA2 regions for the analyses of c-Fos expression following social novelty exposure (Figure 6A-O; Materials and Methods, page 8, lines 14-15). We additionally revised the figure panels and added detailed neuroanatomical information, including the anatomical coordinates analyzed in the present study (Figure 6 A-O; Materials and methods in p. 9, line 21-p. 10, line 4).

Response 3-2:

As requested, we have added quantitative analyses of c-Fos-positive cells in the dorsal CA2 region (A/P: -1.94mm from bregma). As the reviewer correctly noted, previous studies have reported an important role for the dorsal CA2 in social novelty recognition; however, these findings do not preclude the possible involvement of the ventral CA2 in social novelty-related processing. Accordingly, in the revised manuscript we now provide quantitative comparisons of c-Fos expression in both the dorsal and ventral CA2 (A/P: -2.54 mm from bregma) following novel mouse exposure, allowing a more comprehensive evaluation of CA2 subregional contributions to social novelty-associated neuronal activation. These new findings have been added as Figure 6 and described in the revised Results section (Results; p. 17, line 19- p. 18, line 22).

In addition, we performed separate analyses of both the dorsal (A/P: -1.94 mm from bregma) and ventral DG regions (A/P: -2.54 mm from bregma). The dentate gyrus (DG) has been implicated in novelty recognition related to environmental and object information. Furthermore, a recent study by Luo et al. (2025) reported functionally distinct projections from the supramammillary nucleus (SuM) to the dorsal and ventral DG, in which projections to the dorsal DG were associated with event memory processing, whereas projections to the ventral DG were linked to affective processing. Based on these findings, we examined neuronal activation in both the dorsal and ventral DG following novel mouse exposure in the present study. Control mice showed increased neuronal activation selectively in the ventral DG following exposure to a novel mouse, whereas this increase was not observed in either MS or Caps2-/- mice. These new findings have been added as Figure 7 and described in the revised Results section (Results; p. 19, line 1-23).

Major 4:

The phrase "SuM-CA2 pathway" should be used more cautiously. As currently written, it implies that the present study directly examined this pathway, which it did not, and is therefore potentially misleading. While previous studies suggest that the SuM-CA2 pathway plays an important role in social memory, the present study assessed c-Fos expression in the SuM and CA2 independently. Consequently, it remains unclear whether c-Fos expression in SuM neurons led to c-Fos induction in CA2 neurons. The SuM projects to regions other than the CA2 and there are also reciprocal CA2-SuM projections. Accordingly, the statement on p. 14-"These findings demonstrate that deficits in social novelty recognition in both models are accompanied by a failure to activate the SuM-CA2 pathway"-should be tempered to more accurately reflect the data.

Response 4:

We agree with the reviewer that our previous use of the phrase "SuM-CA2 pathway" was an overstatement. In the present study, we independently evaluated c-Fos expression in the SuM and hippocampal regions including CA2 and DG, and therefore our current data do not demonstrate a direct causal relationship or pathway-specific activation between the SuM and hippocampal CA2.

To more accurately reflect our findings and address the reviewer's concerns, we made the following specific revisions.

Tempered claims and terminology:

Throughout the manuscript, we removed statements implying direct "pathway" activation. In addition, we carefully revised the Abstract, Results, and Discussion sections to avoid use of the term "SuM-CA2 pathway" and instead adopted more precise and descriptive language regarding activity changes in each brain region.

Clarification of our core findings:

In the present study, we describe the histological findings that, in two distinct models (MS and Caps2-KO mice), impairments in social novelty recognition in adult male mice were accompanied by a concurrent reduction in social novelty-dependent neuronal activation in the SuM and hippocampal regions including CA2 and DG, regions implicated in social novelty recognition. Based on these observations, we added a new section to the Discussion addressing the concurrent alterations in neuronal activity observed in the SuM and hippocampus. In this section, we revised the interpretation of our findings to provide a more cautious and balanced discussion of the results (Discussion; p. 25, line 21-p. 26, line 22).

Cautious formulation of our hypothesis:

The present study did not directly assess functional connectivity or pathway-specific activity between the SuM and hippocampus. Therefore, future circuit-specific studies will be necessary to determine whether functional coordination between these regions is altered in these models. These limitations have been explicitly described in the Limitation section (Limitation; p. 28, line 17-p. 29, line 2).

Major 5-1:

1. GPR54-positive cells were quantified across the entire SuM, whereas c-Fos was quantified within a restricted midline region (0.145 mm²; A/P −2.80 mm). Similarly, lesions were targeted to the midline SuM at approximately A/P −2.50 mm. Additional justification or clarification is needed to explain these differences in anatomical sampling and targeting. Particularly, do these choices have anything to do with selective projection of midline SuM neurons to the CA2? Response 5-1:

As the reviewer correctly pointed out, in the pre-review version of the manuscript, c-Fos-positive cells in the SuM following social novelty exposure were quantified only within a restricted midline region. In response to this concern, we additionally quantified c-Fos-positive cells across a broader anatomical range of the SuM using the same sampling strategy employed for the GPR54 distribution analysis (Figure 1). Specifically, c-Fos expression was analyzed at three anterior-posterior levels (A/P −2.54 mm, −2.80 mm, and −3.08 mm) in both the medial (midline) and bilateral lateral regions. These new findings have been added as Figure 5.

The results showed that social novelty-dependent c-Fos induction was most prominent in the medial region of the SuM across all anterior-posterior levels, whereas no significant increase associated with social novelty exposure was detected in MS or Caps2⁻/⁻ mice. In addition, we examined the relationship between GPR54 and c-Fos expression across these regions. The medial data are presented in Figure 8, whereas the data of lateral region are included as Extended Data Figures 8-1, 8-2, and 8-3.

Because the present study did not directly examine anatomical or functional projections from the SuM to the CA2 or DG, we intentionally avoided causal interpretations regarding the observed c-Fos induction patterns and revised the Discussion accordingly.

Regarding the reviewer's question concerning selective projections to the CA2, previous anatomical and viral-tracing studies demonstrated that CA2-projecting neurons are enriched within the midline/medial SuM (Soussi et al., 2010; Robert et al., 2021). Consistent with these findings, we observed that social novelty-dependent c-Fos induction in the SuM was more prominent in the midline region than in the lateral regions.

Major 5-2:

2. The GPR54 data are interesting but they are far from conclusive. It would be good to temper the discussion of these findings and to suggest how establishing causality could be achieved.

Response 5-2:

We thank the reviewer for this important comment and fully agree with this concern. Therefore, further studies will be necessary to characterize the GPR54-expressing SuM cells in greater detail, including their molecular identity, neuronal subtype, and projection targets.

In addition, further investigation will be required to clarify the biological significance of the reduced number of GPR54-expressing cells observed in MS mice and the increased number observed in Caps2⁻/⁻ mice, as well as potential alterations in their neuronal properties.

We also agree that the present findings do not establish a causal relationship underlying the reduced SuM neuronal activation observed in MS and Caps2⁻/⁻ mice. Accordingly, we revised the Discussion to provide a more cautious interpretation of these findings and added proposals for future studies regarding GPR54-expressing SuM neurons (Discussion; p. 22, line 6-p. 23, line 17).

Minor Issues • Cell counts should be expressed as /mm2 or /um2 instead of /0.145 mm2 or /104 um2 Response 6:

We appreciate the reviewer for this helpful suggestion. We have updated all cell count units to "/mm²" throughout the entire manuscript and all corresponding figures (Figure 1, 3, 5-8, Extended data, Figure 8-1, 8-2, 8-3). • p. 2: Although the study shows that maternal separation and Caps2 deletion reduce c-Fos expression... it does not demonstrate a complete absence of c-Fos induction. To support such a claim, control groups without social interaction would be required. I recommend rephrasing "no c-Fos induction." Response 7:

We agree with the reviewer's comment. To address this concern, we performed additional control experiments including no-social-interaction conditions and quantitatively compared c-Fos expression in the SuM, CA2, and DG with and without exposure to a socially novel conspecific (Figures 5, 6, 7, and 8, Extended data, Figure 8-1, 8-2, 8-3). We also described the procedures used to generate the control groups without social novelty exposure for c-Fos analysis in a newly added section of the Materials and Methods (Materials and Methods; p. 11, lines 12-24).

Based on these additional analyses, we revised the wording in the Significant Statement to avoid implying a complete absence of c-Fos induction. The revised Significant Statement is shown below.

Revised Significant Statement: "We tested whether the supramammillary nucleus (SuM) is altered in neurodevelopmental disorder models with impaired social novelty recognition. In control mice, exposure to a novel conspecific robustly induced c-Fos expression in the SuM, hippocampal CA2, and DG, whereas maternal separation (MS) and Caps2⁻/⁻ mice showed reduced activation in these regions. The two models also exhibited divergent changes in SuM GPR54 expression, with reduced expression in MS mice and an increased number of GPR54-expressing cells in Caps2⁻/⁻ mice. These findings suggest that social novelty deficits are associated with reduced activation in the SuM, CA2, and DG across distinct models, and that differences in GPR54 expression may reflect the involvement of distinct underlying molecular alterations across mouse models with impaired social novelty recognition but different etiologies." • p. 6: Remove the "J" from "C57BL/6J," as this designation specifically indicates mice sourced from The Jackson Laboratory.

Response 8:

We appreciate this correction. We have removed the "J" from "C57BL/6" on page 6 and throughout the entire manuscript (Materials and methods; p. 6, lines 17-18).

We sincerely appreciate the reviewers' insightful comments and believe that these revisions have substantially strengthened the manuscript. We hope that the revised manuscript is now suitable for publication in eNeuro.

## References

[B1] Banerjee S, Riordan M, Bhat MA (2014) Genetic aspects of autism spectrum disorders: insights from animal models. Front Cell Neurosci 8:58. 10.3389/fncel.2014.00058 24605088 PMC3932417

[B2] Boyle LM, Sheng W, Villegas A, Sahai R, Irfan S, Lee HJ, Young WS, Leroy F, Siegelbaum SA (2025) The ventral CA2 region of the hippocampus and its differential contributions to social memory and social aggression. Cell Rep 44:115714. 10.1016/j.celrep.2025.115714 40372914 PMC12308836

[B3] Chan YM, Broder-Fingert S, Wong KM, Seminara SB (2009) Kisspeptin/Gpr54-independent gonadotrophin-releasing hormone activity in Kiss1 and Gpr54 mutant mice. J Neuroendocrinol 21:1015–1023. 10.1111/j.1365-2826.2009.01926.x 19840236 PMC2789182

[B4] Chen P, Hong W (2018) Neural circuit mechanisms of social behavior. Neuron 98:16–30. 10.1016/j.neuron.2018.02.026 29621486 PMC6028944

[B5] Chen S, et al. (2020) A hypothalamic novelty signal modulates hippocampal memory. Nature 586:270–274. 10.1038/s41586-020-2771-1 32999460

[B6] Choi GB, Dong HW, Murphy AJ, Valenzuela DM, Yancopoulos GD, Swanson LW, Anderson DJ (2005) Lhx6 delineates a pathway mediating innate reproductive behaviors from the amygdala to the hypothalamus. Neuron 46:647–660. 10.1016/j.neuron.2005.04.011 15944132

[B7] Clarke S, Chen Z, Hsu MS, Hill RG, Pintar JE, Kitchen I (2003) Nociceptin/orphanin FQ knockout mice display up-regulation of the opioid receptor-like 1 receptor and alterations in opioid receptor expression in the brain. Neuroscience 117:157–168. 10.1016/S0306-4522(02)00750-9 12605902

[B8] Clarkson J, d'Anglemont de Tassigny X, Moreno AS, Colledge WH, Herbison AE (2008) Kisspeptin-GPR54 signaling is essential for preovulatory gonadotropin-releasing hormone neuron activation and the luteinizing hormone surge. J Neurosci 28:8691–8697. 10.1523/JNEUROSCI.1775-08.2008 18753370 PMC6670827

[B9] Clarkson J, Herbison AE (2006) Postnatal development of kisspeptin neurons in mouse hypothalamus; sexual dimorphism and projections to gonadotropin-releasing hormone neurons. Endocrinology 147:5817–5825. 10.1210/en.2006-0787 16959837 PMC6098691

[B10] de Roux N, Genin E, Carel JC, Matsuda F, Chaussain JL, Milgrom E (2003) Hypogonadotropic hypogonadism due to loss of function of the Kiss1-derived peptide receptor GPR54. Proc Natl Acad Sci U S A 100:10972–10976. 10.1073/pnas.1834399100 12944565 PMC196911

[B11] Diethorn EJ, Gould E (2022) Postnatal development of hippocampal CA2 structure and function during the emergence of social recognition of peers. Hippocampus 33:208–222. 10.1002/hipo.23476 36309963 PMC10028396

[B12] Ellegood J, Crawley JN (2015) Behavioral and neuroanatomical phenotypes in mouse models of autism. Neurotherapeutics 12:521–533. 10.1007/s13311-015-0360-z 26036957 PMC4489953

[B13] Han SK, Gottsch ML, Lee KJ, Popa SM, Smith JT, Jakawich SK, Clifton DK, Steiner RA, Herbison AE (2005) Activation of gonadotropin-releasing hormone neurons by kisspeptin as a neuroendocrine switch for the onset of puberty. J Neurosci 25:11349–11356. 10.1523/JNEUROSCI.3328-05.2005 16339030 PMC6725899

[B14] Herbison AE, Xd dT, Doran J, Colledge WH (2010) Distribution and postnatal development of *Gpr54* gene expression in mouse brain and gonadotropin-releasing hormone neurons. Endocrinology 151:312–321. 10.1210/en.2009-0552 19966188

[B15] Higo S, Honda S, Iijima N, Ozawa H (2016) Mapping of kisspeptin receptor mRNA in the whole rat brain and its co-localisation with oxytocin in the paraventricular nucleus. J Neuroendocrinol 28:e12356. 10.1111/jne.12356 26709462

[B16] Hitti FL, Siegelbaum SA (2014) The hippocampal CA2 region is essential for social memory. Nature 508:88–92. 10.1038/nature13028 24572357 PMC4000264

[B17] Iguchi H, et al. (2024) Calcium-dependent activator protein for secretion 2 is involved in dopamine release in mouse midbrain neurons. Front Mol Neurosci 17:1444629. 10.3389/fnmol.2024.1444629 39092202 PMC11291307

[B18] Kauffman AS, et al. (2007) The kisspeptin receptor GPR54 is required for sexual differentiation of the brain and behavior. J Neurosci 27:8826–8835. 10.1523/JNEUROSCI.2099-07.2007 17699664 PMC6672184

[B19] Kaushik G, Zarbalis KS (2016) Prenatal neurogenesis in autism spectrum disorders. Front Chem 4:12. 10.3389/fchem.2016.00012 27014681 PMC4791366

[B20] Kinsey-Jones JS, Li XF, Knox AM, Wilkinson ES, Zhu XL, Chaudhary AA, Milligan SR, Lightman SL, O'Byrne KT (2009) Down-regulation of hypothalamic kisspeptin and its receptor, Kiss1r, mRNA expression is associated with stress-induced suppression of luteinising hormone secretion in the female rat. J Neuroendocrinol 21:20–29. 10.1111/j.1365-2826.2008.01807.x 19094090

[B21] Kotani M, et al. (2001) The metastasis suppressor gene KiSS-1 encodes kisspeptins, the natural ligands of the orphan G protein-coupled receptor GPR54. J Biol Chem 276:34631–34636. 10.1074/jbc.M104847200 11457843

[B22] Lapatto R, Pallais JC, Zhang D, Chan YM, Mahan A, Cerrato F, Le WW, Hoffman GE, Seminara SB (2007) Kiss1-/- mice exhibit more variable hypogonadism than Gpr54-/- mice. Endocrinology 148:4927–4936. 10.1210/en.2007-0078 17595229

[B23] Liu D, Diorio J, Tannenbaum B, Caldji C, Francis D, Freedman A, Sharma S, Pearson D, Plotsky PM, Meaney MJ (1997) Maternal care, hippocampal glucocorticoid receptors, and hypothalamic-pituitary-adrenal responses to stress. Science 277:1659–1662. 10.1126/science.277.5332.1659 9287218

[B24] Liu X, Lee K, Herbison AE (2008) Kisspeptin excites gonadotropin-releasing hormone neurons through a phospholipase C/calcium-dependent pathway regulating multiple ion channels. Endocrinology 149:4605–4614. 10.1210/en.2008-0321 18483150 PMC6116891

[B25] Maglóczky Z, Acsády L, Freund TF (1994) Principal cells are the postsynaptic targets of supramammillary afferents in the hippocampus of the rat. Hippocampus 4:322–334. 10.1002/hipo.450040316 7531093

[B26] Mathis A, Mamidanna P, Cury KM, Abe T, Murthy VN, Mathis MW, Bethge M (2018) DeepLabCut: markerless pose estimation of user-defined body parts with deep learning. Nat Neurosci 21:1281–1289. 10.1038/s41593-018-0209-y 30127430

[B27] Naninck EF, Hoeijmakers L, Kakava-Georgiadou N, Meesters A, Lazic SE, Lucassen PJ, Korosi A (2015) Chronic early life stress alters developmental and adult neurogenesis and impairs cognitive function in mice. Hippocampus 25:309–328. 10.1002/hipo.22374 25269685

[B28] Ohtaki T, et al. (2001) Metastasis suppressor gene KISS-1 encodes peptide ligand of a G-protein-coupled receptor. Nature 411:613–617. 10.1038/35079135 11385580

[B29] Okuyama T, Kitamura T, Roy DS, Itohara S, Tonegawa S (2016) Ventral CA1 neurons store social memory. Science 353:1536–1541. 10.1126/science.aaf7003 27708103 PMC5493325

[B30] O'Mahony SM, Hyland NP, Dinan TG, Cryan JF (2011) Maternal separation as a model of brain-gut axis dysfunction. Psychopharmacology (Berl) 214:71–88. 10.1007/s00213-010-2010-9 20886335

[B31] Ozawa H (2021) Kisspeptin neurons as an integration center of reproductive regulation: observation of reproductive function based on a new concept of reproductive regulatory nervous system. Reprod Med Biol 21:e12419. 10.1002/rmb2.12419 34934400 PMC8656200

[B32] Pan WX, McNaughton N (2002) The role of the medial supramammillary nucleus in the control of hippocampal theta activity and behaviour in rats. Eur J Neurosci 16:1797–1809. 10.1046/j.1460-9568.2002.02267.x 12431233

[B33] Pan WX, McNaughton N (2004) The supramammillary area: its organization, functions and relationship to the hippocampus. Prog Neurobiol 74:127–166. 10.1016/j.pneurobio.2004.09.003 15556285

[B34] Paxinos G, Franklin KBJ (2013) *Paxinos and Franklin's the mouse brain in stereotaxic coordinates*, Ed. 4. San Diego: Academic Press.

[B35] Plotsky PM, Meaney MJ (1993) Early, postnatal experience alters hypothalamic corticotropin-releasing factor (CRF) mRNA, median eminence CRF content and stress-induced release in adult rats. Brain Res Mol Brain Res 18:195–200. 10.1016/0169-328X(93)90189-V 8497182

[B36] Rein B, Ma K, Yan Z (2020) A standardized social preference protocol for measuring social deficits in mouse models of autism. Nat Protoc 15:3464–3477. 10.1038/s41596-020-0382-9 32895524 PMC8103520

[B37] Rizzi-Wise CA, Wang DV (2021) Putting together pieces of the lateral septum: multifaceted functions and its neural pathways. eNeuro 8:ENEURO 0315-21 1-11. 10.1523/ENEURO.0315-21.2021 PMC8647703 34764187

[B38] Rombaut C, Roura-Martinez D, Lepolard C, Gascon E (2023) Brief and long maternal separation in C57Bl6J mice: behavioral consequences for the dam and the offspring. Front Behav Neurosci 17:1269866. 10.3389/fnbeh.2023.1269866 37936649 PMC10626007

[B39] Sadakata T, Mizoguchi A, Sato Y, Katoh-Semba R, Fukuda M, Mikoshiba K, Furuichi T (2004) The secretory granule-associated protein CAPS2 regulates neurotrophin release and cell survival. J Neurosci 24:43–52. 10.1523/JNEUROSCI.2528-03.2004 14715936 PMC6729559

[B40] Sadakata T, et al. (2007) Autistic-like phenotypes in Cadps2-knockout mice and aberrant CADPS2 splicing in autistic patients. J Clin Invest 117:931–943. 10.1172/JCI29031 17380209 PMC1821065

[B41] Sadakata T, Shinoda Y, Oka M, Sekine Y, Sato Y, Saruta C, Miwa H, Tanaka M, Itohara S, Furuichi T (2012) Reduced axonal localization of a Caps2 splice variant impairs axonal release of BDNF and causes autistic-like behavior in mice. Proc Natl Acad Sci U S A 109:21104–21109. 10.1073/pnas.1210055109 23213205 PMC3529019

[B42] Seminara SB, et al. (2003) The GPR54 gene as a regulator of puberty. N Engl J Med 349:1614–1627. 10.1056/NEJMoa035322 14573733

[B43] Shin HS, Choi SM, Lee SH, Moon HJ, Jung EM (2023) A novel early life stress model affects brain development and behavior in mice. Int J Mol Sci 24:4688. 10.3390/ijms24054688 36902120 PMC10002977

[B44] Shinoda Y, Sadakata T, Nakao K, Katoh-Semba R, Kinameri E, Furuya A, Yanagawa Y, Hirase H, Furuichi T (2011) Calcium-dependent activator protein for secretion 2 (CAPS2) promotes BDNF secretion and is critical for the development of GABAergic interneuron network. Proc Natl Acad Sci U S A 108:373–378. 10.1073/pnas.1012220108 21173225 PMC3017206

[B45] Szentkirályi-Tóth S, et al. (2025) Estrogen-regulated lateral septal kisspeptin neurons abundantly project to GnRH neurons and the hypothalamic supramammillary nucleus. J Neurosci 45:e1307242024. 10.1523/JNEUROSCI.1307-24.2024 39746822 PMC11841763

[B46] Szereszewski JM, Pampillo M, Ahow MR, Offermanns S, Bhattacharya M, Babwah AV (2010) GPR54 regulates ERK1/2 activity and hypothalamic gene expression in a Gα(q/11) and β-arrestin-dependent manner. PLoS One 5:e12964. 10.1371/journal.pone.0012964 20886089 PMC2944883

[B47] Takumi K, Iijima N, Higo S, Ozawa H (2012) Immunohistochemical analysis of the colocalization of corticotropin-releasing hormone receptor and glucocorticoid receptor in kisspeptin neurons in the hypothalamus of female rats. Neurosci Lett 531:40–45. 10.1016/j.neulet.2012.10.010 23069671

[B48] Thirtamara Rajamani K, Barbier M, Lefevre A, Niblo K, Cordero N, Netser S, Grinevich V, Wagner S, Harony-Nicolas H (2024) Oxytocin activity in the paraventricular and supramammillary nuclei of the hypothalamus is essential for social recognition memory in rats. Mol Psychiatry 29:412–424. 10.1038/s41380-023-02336-0 38052983 PMC11116117

[B49] Trivedi PG, Yu H, Trumbauer M, Chen H, Van der Ploeg LH, Guan X (2001) Differential regulation of neuropeptide Y receptors in the brains of NPY knock-out mice. Peptides 22:395–403. 10.1016/S0196-9781(01)00349-7 11287094

[B50] Vertes RP (2015) Major diencephalic inputs to the hippocampus: supramammillary nucleus and nucleus reuniens. Circuitry and function. In: *The connected hippocampus. Prog brain Res. 219* (O'Mara S, Tsanov M, eds), pp 121–144. Amsterdam: Elsevier B.V. 10.1016/bs.pbr.2015.03.008 PMC4961211 26072237

[B51] Wyss JM, Swanson LW, Cowan WM (1979) Evidence for an input to the molecular layer and the stratum granulosum of the dentate gyrus from the supramammillary region of the hypothalamus. Anat Embryol (Berl) 156:165–176. 10.1007/BF00300012 464319

[B52] Yamanaka O, Takeuchi R (2018) UMA tracker: an intuitive image-based tracking platform. J Exp Biol 221:jeb182469. 10.1242/jeb.182469 29954834

[B53] Zhang C, Roepke TA, Kelly MJ, Rønnekleiv OK (2008) Kisspeptin depolarizes gonadotropin-releasing hormone neurons through activation of TRPC-like cationic channels. J Neurosci 28:4423–4434. 10.1523/JNEUROSCI.5352-07.2008 18434521 PMC6670958

[B54] Zhang L, Hernández VS, Zetter MA, Hernández-Pérez OR, Hernández-González R, Camacho-Arroyo I, Eiden LE, Millar RP (2025) Kisspeptin fiber and receptor distribution analysis suggests its potential role in central sensorial processing and behavioral state control. J Neuroendocrinol 37:e70007. 10.1111/jne.70007 40065551 PMC12045677

