# Supplementary figures and images for: Reduced SuM Activation Accompanies Impaired Social Novelty Recognition in Mouse Models of Neurodevelopmental Disorders
Source: eNeuro. 2026 Jul 7;13(7):ENEURO.0440-25.2026. doi: 10.1523/ENEURO.0440-25.2026 (PMC13349467; doi:10.1523/ENEURO.0440-25.2026)

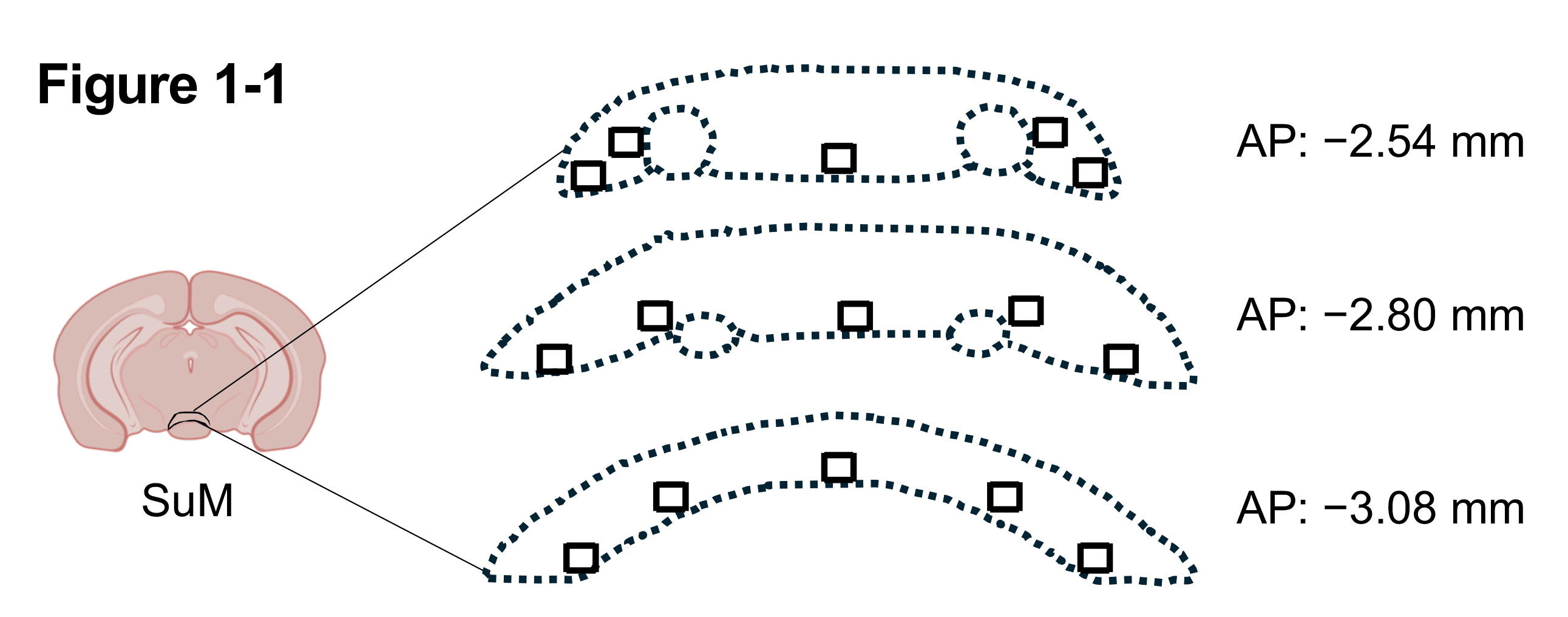

Supplement: Figure 1-1 — Schematic diagram of the mouse coronal section indicating the position and outline of SuM at A/P: −2.54, −2.80, and −3.08 mm from bregma. The five frames in the SuM indicate the subregions where GPR54⁺ cells were counted at P35 and P56. Download Figure 1-1, TIF file. [file eneuro-13-ENEURO.0440-25.2026-s002.tif]

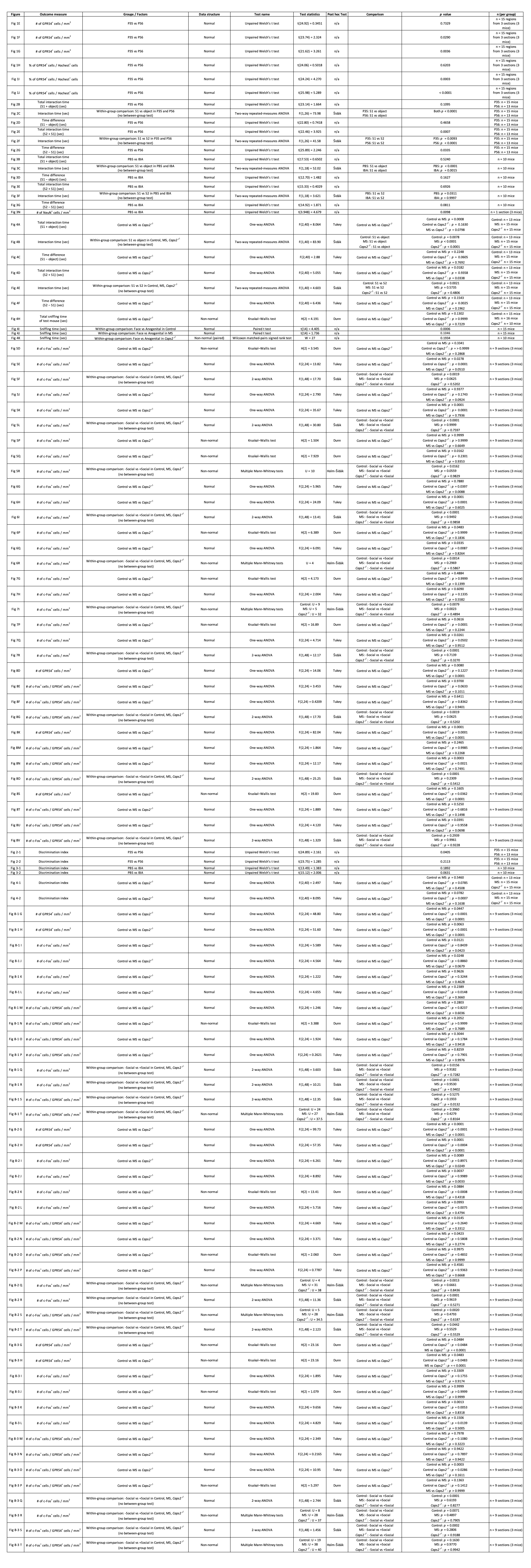

Supplement: Figure 1-2 — Statistical analyses supporting Figures 1–8 and associated Extended Data figures. Download Figure 1-2, TIF file. [file eneuro-13-ENEURO.0440-25.2026-s003.tif]

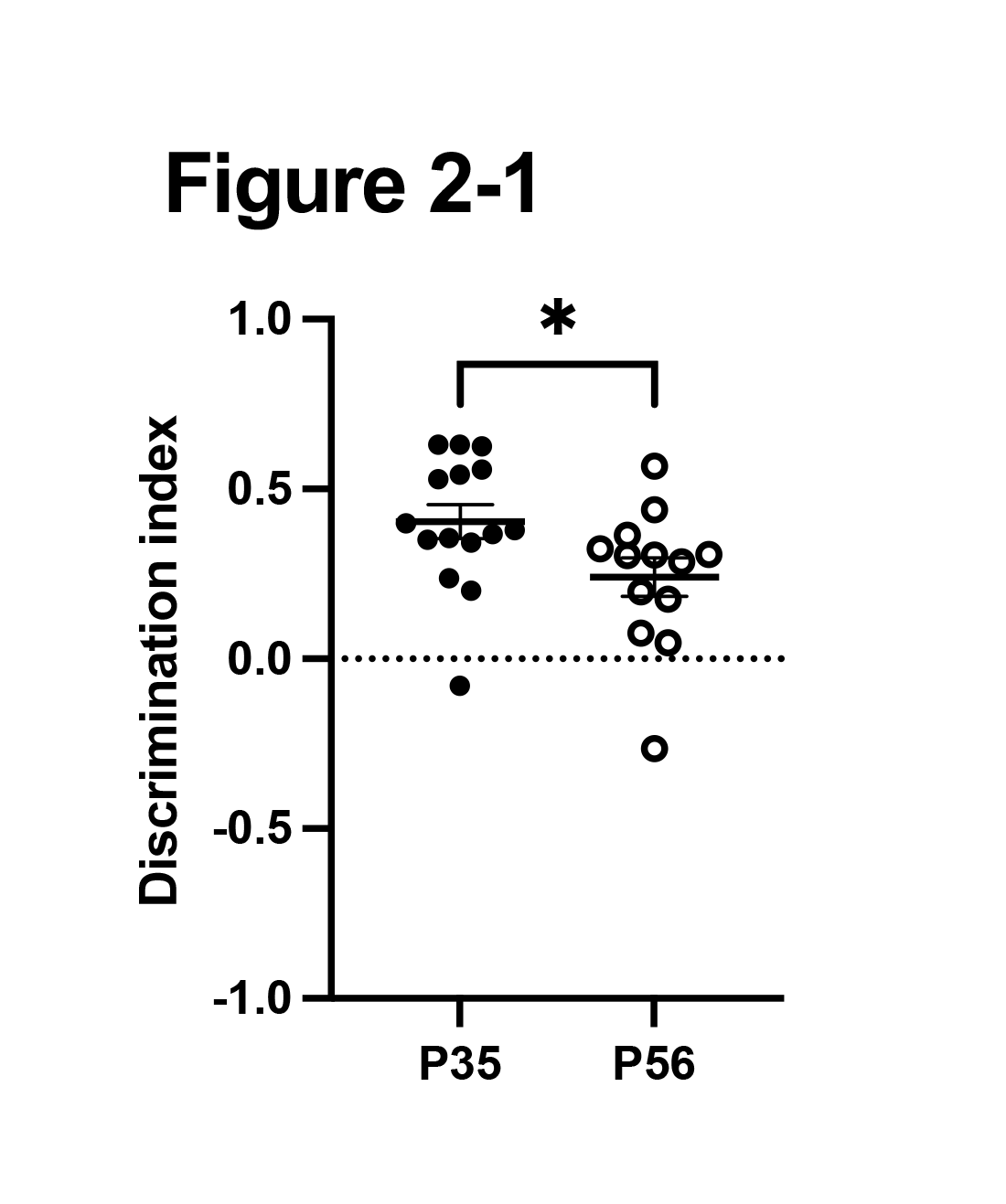

Supplement: Figure 2-1 — Discrimination index for the sociability phase at P35 and P56 mice. P35 mice exhibited a stronger preference for S1 than P56 mice (n = 15 mice for P35, 13 mice for P56). *p < 0.05. See Figure 1-2 for detailed statistical information. Download Figure 2-1, TIF file. [file eneuro-13-ENEURO.0440-25.2026-s004.tif]

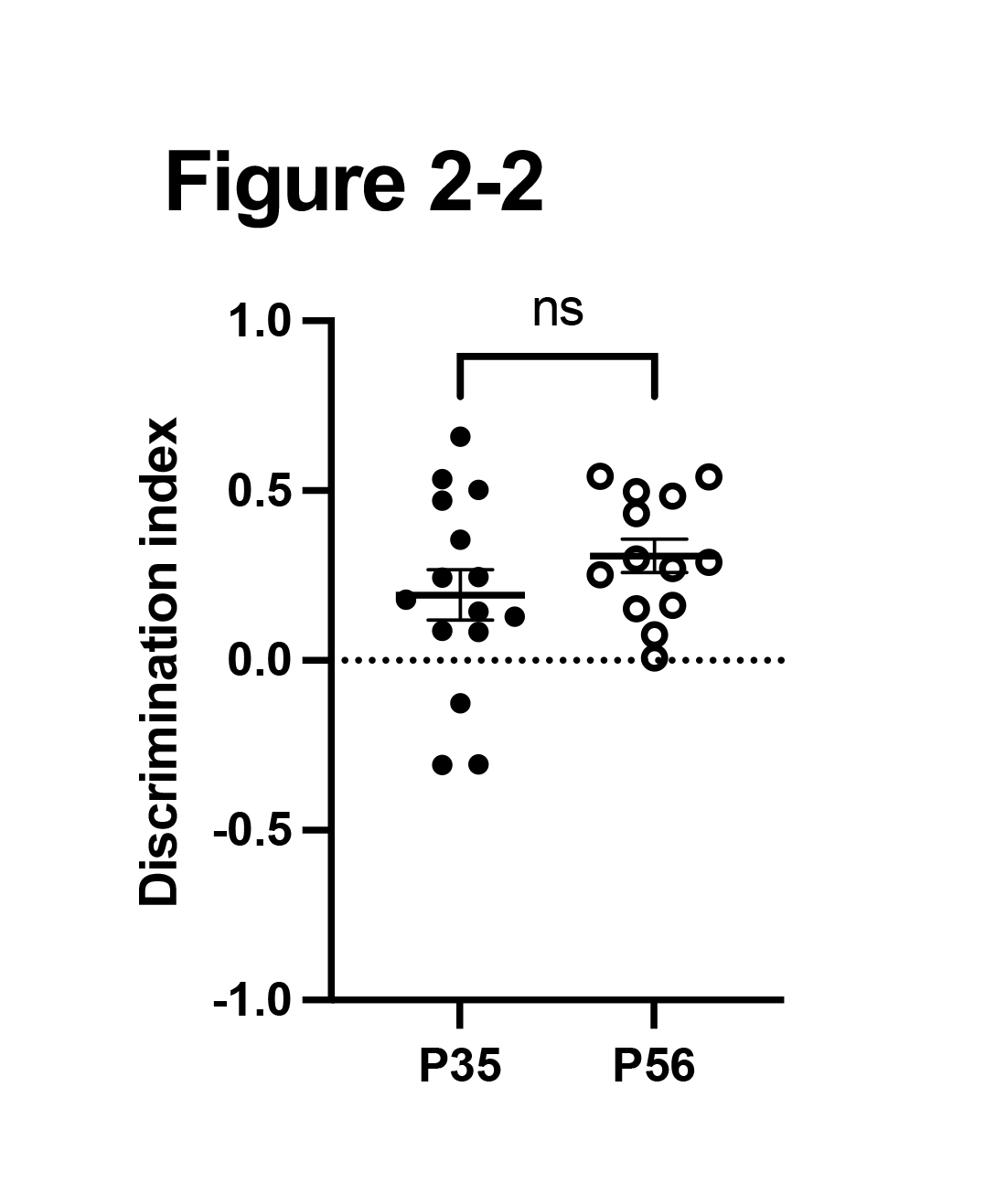

Supplement: Figure 2-2 — Discrimination index for the social novelty phase at P35 and P56 mice. No significant difference of the preference toward S2 between P35 and P56 mice (n = 15 mice for P35, 13 mice for P56). ns, not significant. See Figure 1-2 for detailed statistical information. Download Figure 2-2, TIF file. [file eneuro-13-ENEURO.0440-25.2026-s005.tif]

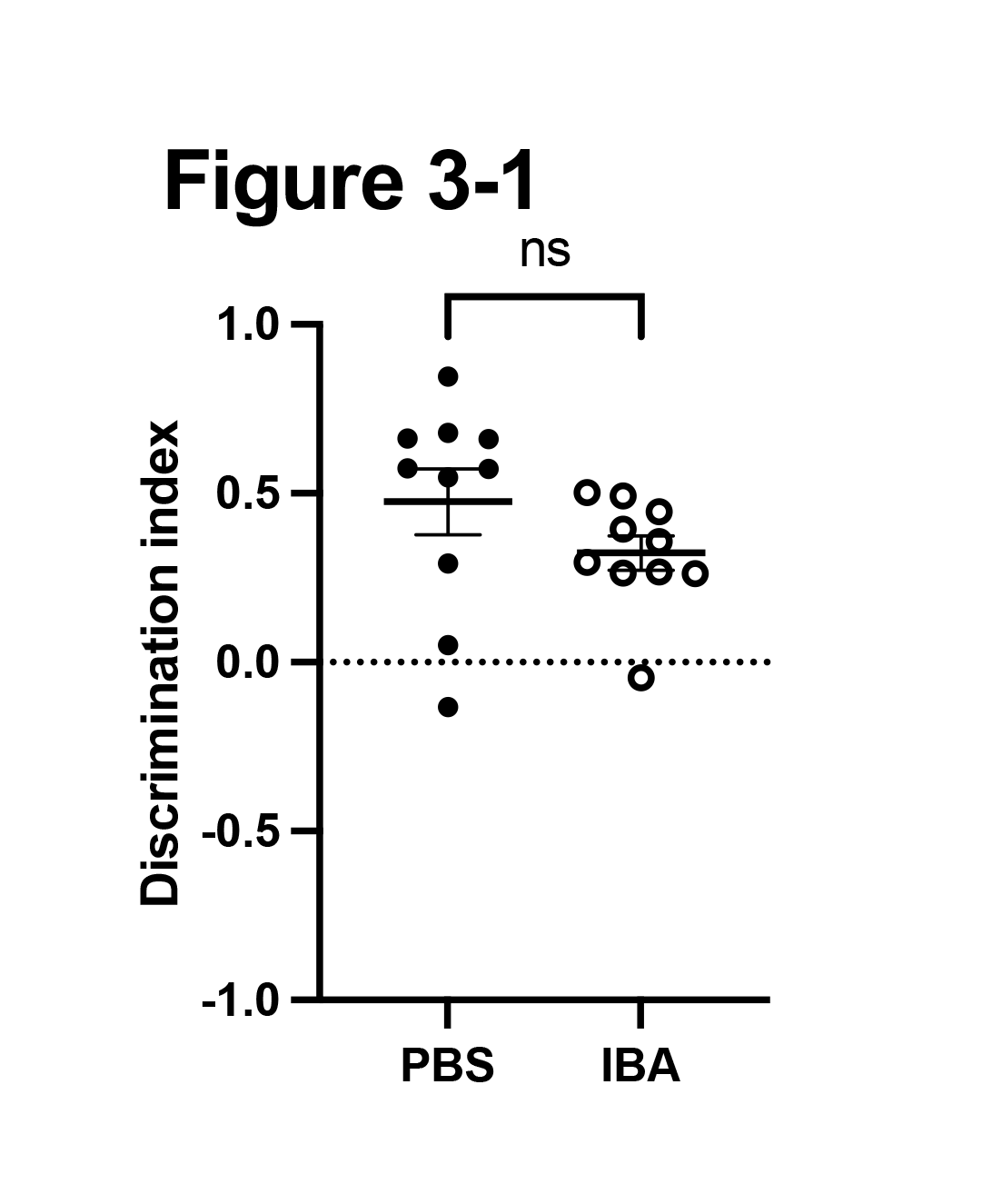

Supplement: Figure 3-1 — Discrimination index for the sociability phase for PBS- and IBA-injected mice. No significant difference of the preference toward S1 between PBS- and IBA-injected mice (n = 10 mice per group). ns., not significant. See Figure 1-2 for detailed statistical information. Download Figure 3-1, TIF file. [file eneuro-13-ENEURO.0440-25.2026-s006.tif]

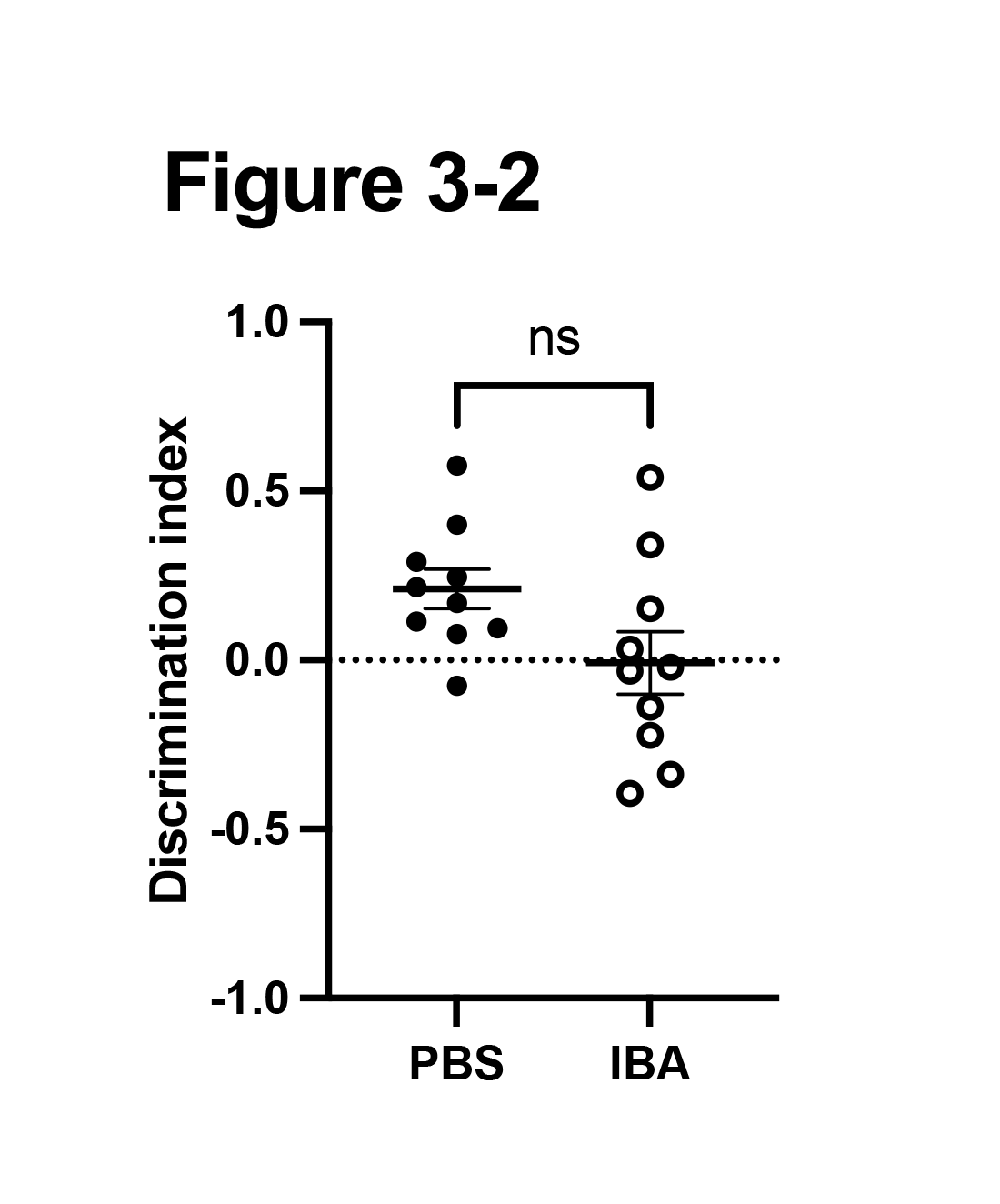

Supplement: Figure 3-2 — Discrimination index for the social novelty phase for PBS- and IBA-injected mice. No significant difference of the preference toward S2 between PBS- and IBA-injected mice (n = 10 mice per group). ns., not significant. See Figure 1-2 for detailed statistical information. Download Figure 3-2, TIF file. [file eneuro-13-ENEURO.0440-25.2026-s007.tif]

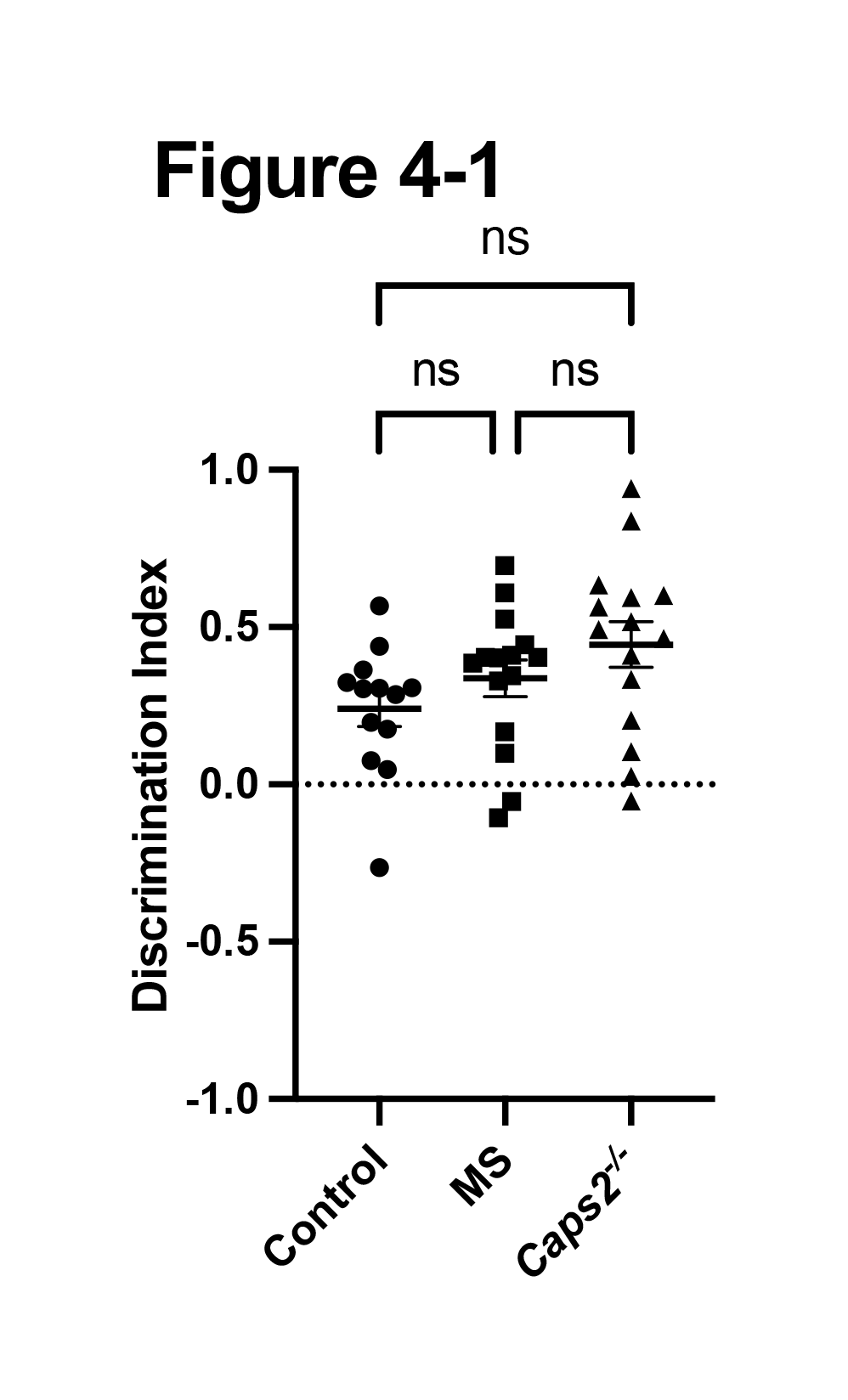

Supplement: Figure 4-1 — Discrimination index for the sociability phase for control, MS and Caps2-/- mice. No significant difference of the preference toward S1 between control, MS, and Caps2-/- mice (n = 13 mice for control, 15 mice for MS, 15 mice for Caps2-/-). ns., not significant. See Figure 1-2 for detailed statistical information. Download Figure 4-1, TIF file. [file eneuro-13-ENEURO.0440-25.2026-s008.tif]

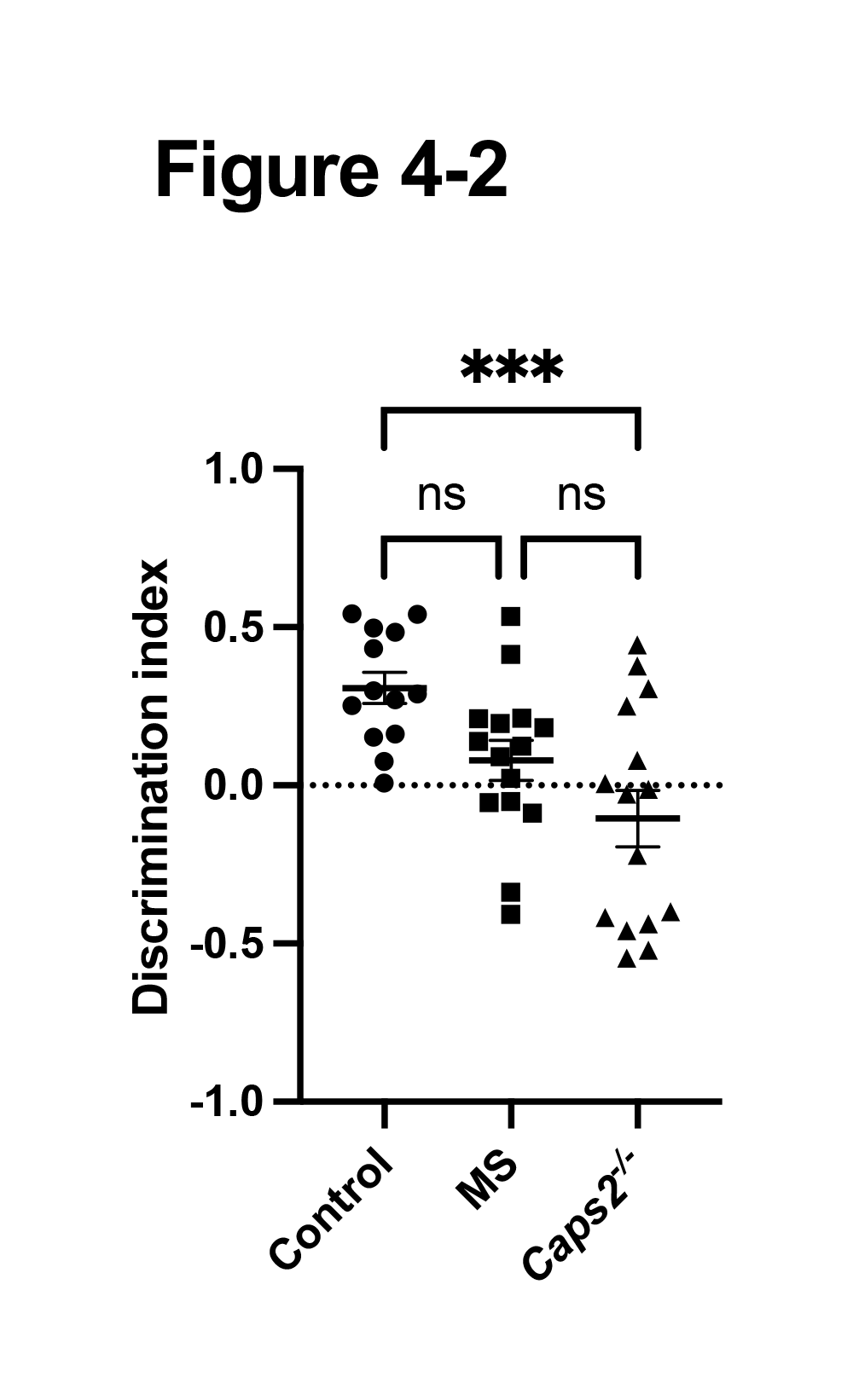

Supplement: Figure 4-2 — Discrimination index for the social novelty phase for control, MS and Caps2-/- mice. Control mice showed significantly stronger preference toward S2 than Caps2-/- mice (n = 13 for control, n = 15 for Caps2-/-), whereas control and MS mice showed a trend but did not reach significance (n = 15 for MS). No significant difference of the preference toward S2 between MS and Caps2-/- mice. *p < 0.05, ns., not significant. See Figure 1-2 for detailed statistical information. Download Figure 4-2, TIF file. [file eneuro-13-ENEURO.0440-25.2026-s009.tif]

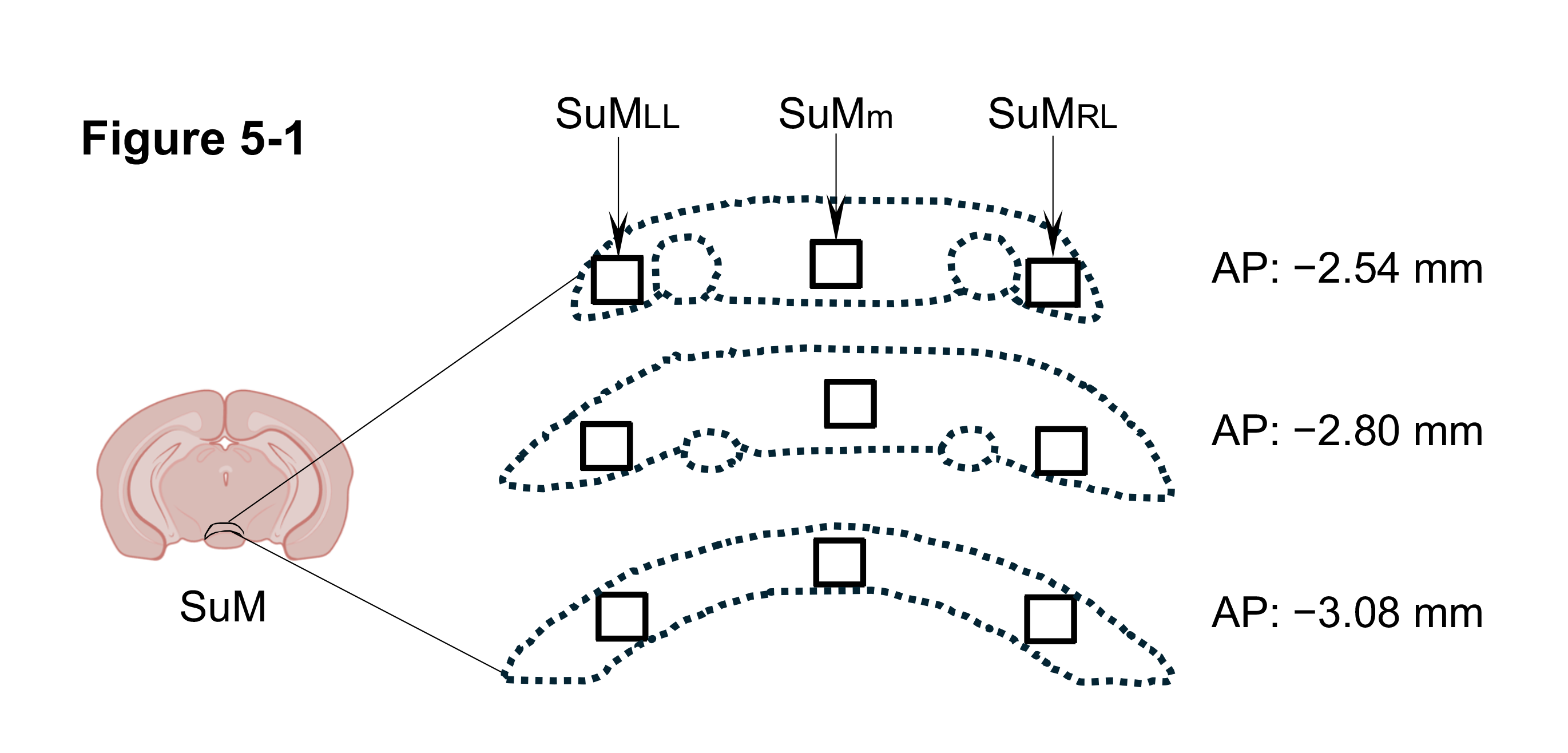

Supplement: Figure 5-1 — Schematic diagrams of mouse coronal sections showing the SuM at A/P –2.54 mm, –2.80 mm, and –3.08 mm from bregma. The three boxed regions within the SuM indicate the left lateral SuM (SuMLL), medial SuM (SuMm), and right lateral SuM (SuMRL) subregions, where c-Fos⁺ and GPR54⁺ cells were quantified at P67. Download Figure 5-1, TIF file. [file eneuro-13-ENEURO.0440-25.2026-s010.tif]

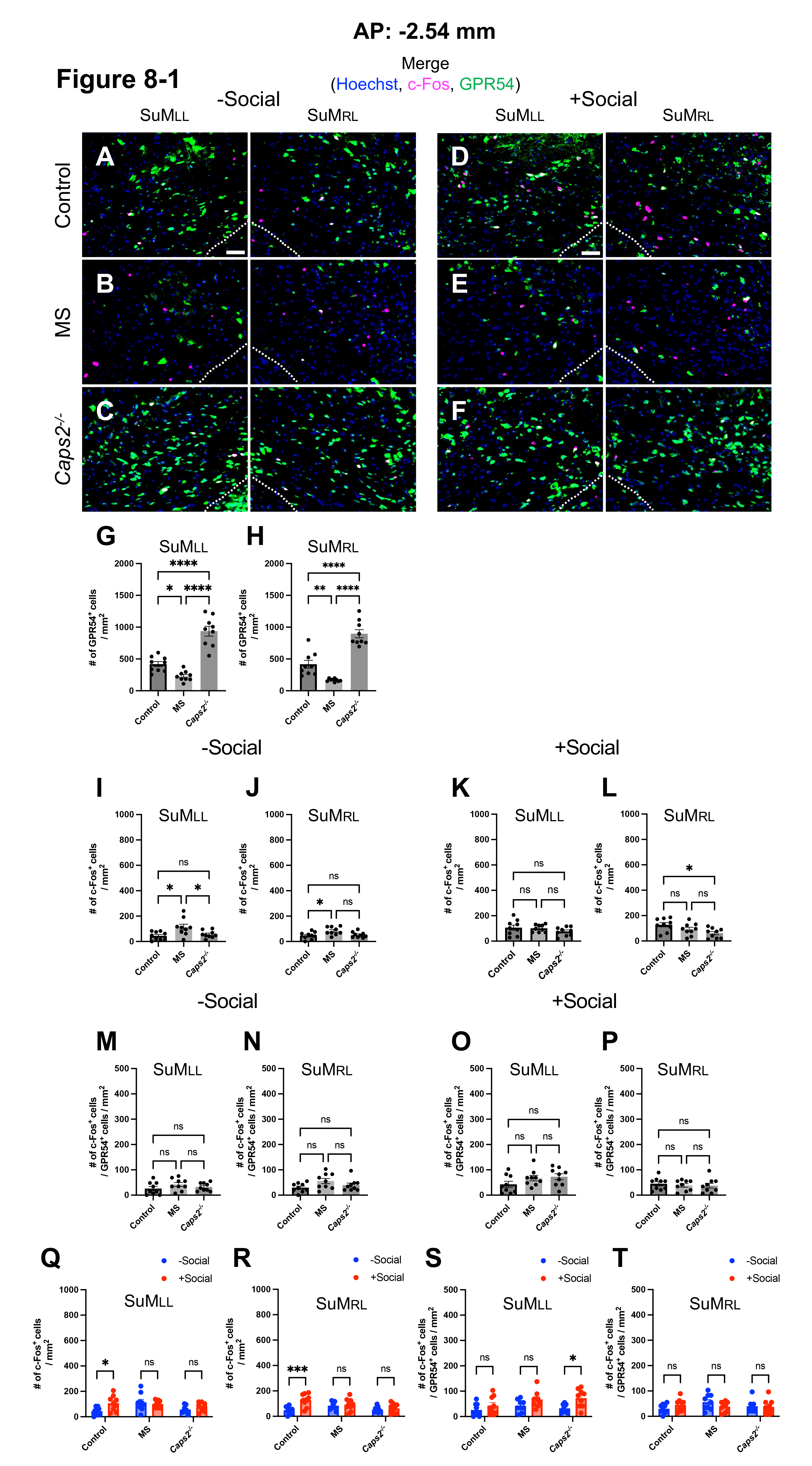

Supplement: Figure 8-1 — A–F, Representative images of merged Hoechst/c-Fos/GPR54 immunofluorescence signals in the left lateral SuM (SuMLL; left images) and right lateral SuM (SuMRL; right images) at A/P –2.54 mm from bregma under the –social (A–C) and +social (D–F) conditions in control (A, D), MS (B, E), and Caps2-/- (C, F) mice. White dotted lines indicate the borders between the SuM and medial mammillary nucleus (MM). Scale bar = 50 μm. G, H, GPR54⁺ cell density in the SuMLL (G) and SuMRL (H). I, J, c-Fos⁺ cell density under the –social condition in the SuMLL (I) and SuMRL (J). K, L, c-Fos⁺ cell density under the +social condition in the SuMLL (K) and SuMRL (L). M, N, c-Fos⁺/GPR54⁺ cell density under the –social condition in the SuMLL (M) and SuMRL (N). O, P, c-Fos⁺/GPR54⁺ cell density under the +social condition in the SuMLL (O) and SuMRL (P). Q, R, Comparison of c-Fos⁺ cell density between the –social and +social conditions in the SuMLL (Q) and SuMRL (R). S, T, Comparison of c-Fos⁺/GPR54⁺ cell density between the –social and +social conditions in the SuMLL (S) and SuMRL (T). n = 3 sections per mouse, 3 mice per group. Filled circles represent values from individual sections. Data are presented as mean ± SEM (G–T). *p < 0.05, **p < 0.01, ***p < 0.001, ****p < 0.0001; ns, not significant. See Figure 1-2 for detailed statistical information. Download Figure 8-1, TIF file. [file eneuro-13-ENEURO.0440-25.2026-s011.tif]

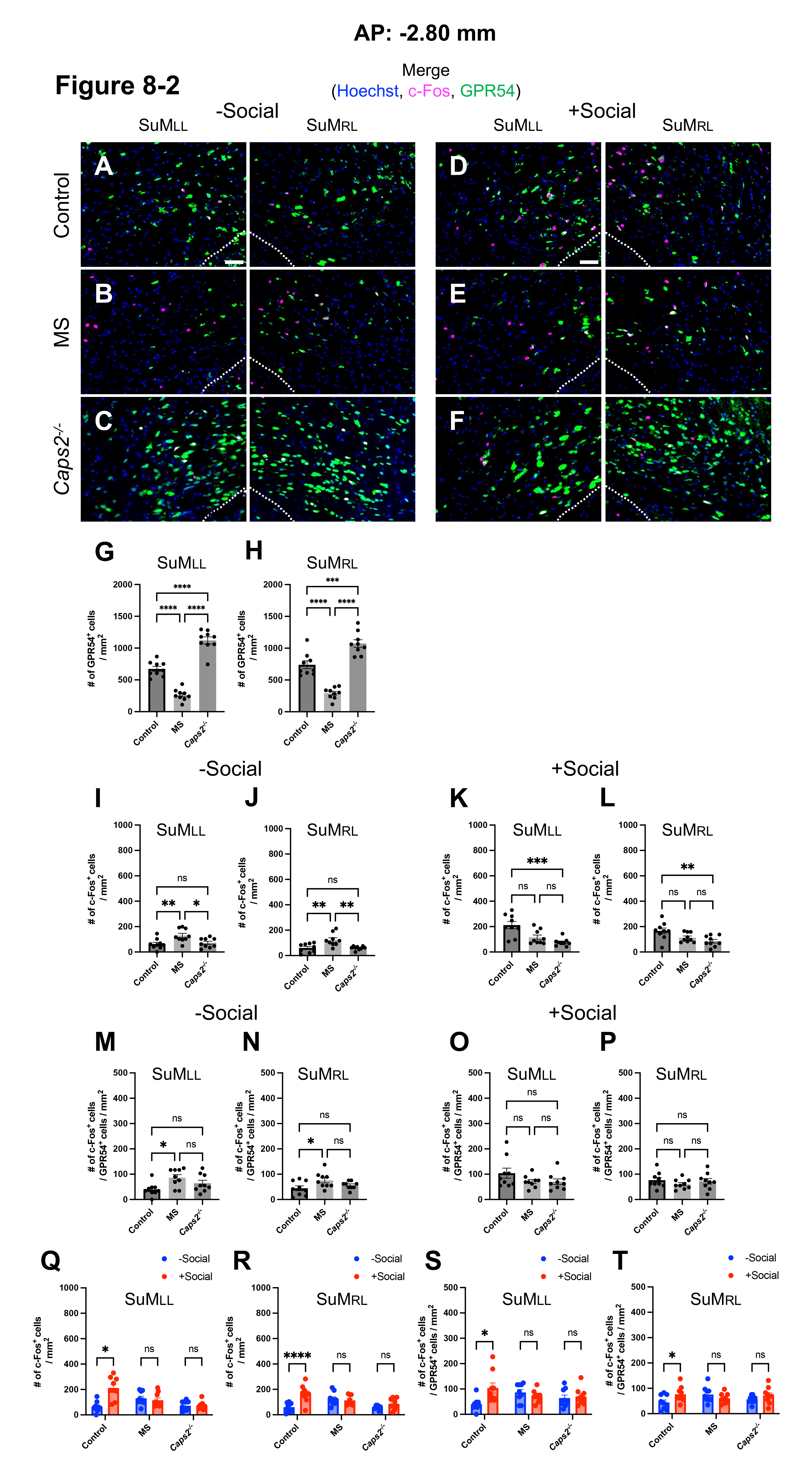

Supplement: Figure 8-2 — A–F, Representative images of merged Hoechst/c-Fos/GPR54 immunofluorescence signals in the left lateral SuM (SuMLL; left images) and right lateral SuM (SuMRL; right images) at A/P –2.80 mm from bregma under the –social (A–C) and +social (D–F) conditions in control (A, D), MS (B, E), and Caps2-/- (C, F) mice. White dotted lines indicate the borders between the SuM and medial mammillary nucleus (MM). Scale bar = 50 μm. G, H, GPR54⁺ cell density in the SuMLL (G) and SuMRL (H). I, J, c-Fos⁺ cell density under the –social condition in the SuMLL (I) and SuMRL (J). K, L, c-Fos⁺ cell density under the +social condition in the SuMLL (K) and SuMRL (L). M, N, c-Fos⁺/GPR54⁺ cell density under the –social condition in the SuMLL (M) and SuMRL (N). O, P, c-Fos⁺/GPR54⁺ cell density under the +social condition in the SuMLL (O) and SuMRL (P). Q, R, Comparison of c-Fos⁺ cell density between the –social and +social conditions in the SuMLL (Q) and SuMRL (R). S, T, Comparison of c-Fos⁺/GPR54⁺ cell density between the –social and +social conditions in the SuMLL (S) and SuMRL (T). n = 3 sections per mouse, 3 mice per group. Filled circles represent values from individual sections. Data are presented as mean ± SEM (G–T). *p < 0.05, **p < 0.01, ***p < 0.001, ****p < 0.0001; ns, not significant. See Figure 1-2 for detailed statistical information. Download Figure 8-2, TIF file. [file eneuro-13-ENEURO.0440-25.2026-s012.tif]

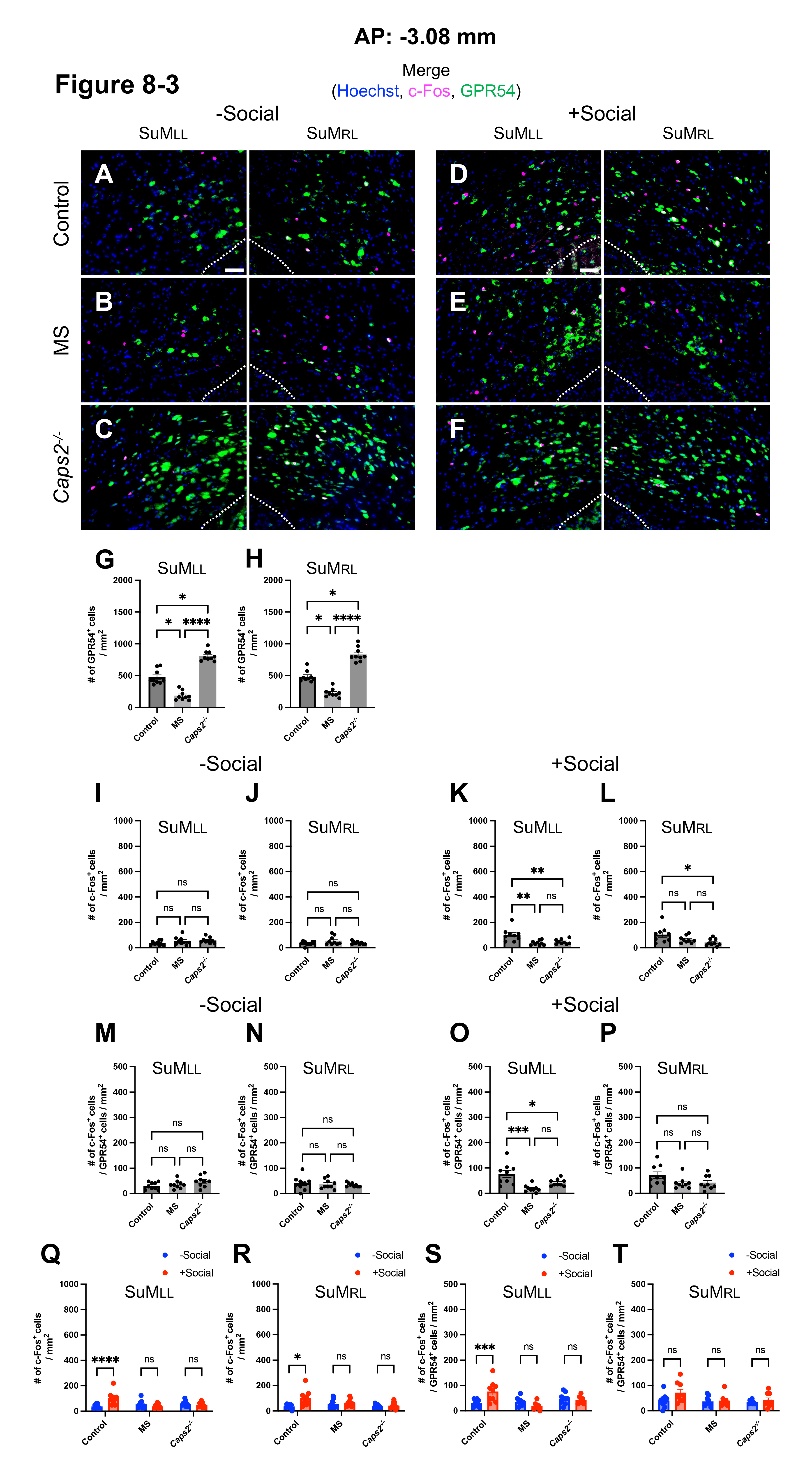

Supplement: Figure 8-3 — A–F, Representative images of merged Hoechst/c-Fos/GPR54 immunofluorescence signals in the left lateral SuM (SuMLL; left images) and right lateral SuM (SuMRL; right images) at A/P –3.08 mm from bregma under the –social (A–C) and +social (D–F) conditions in control (A, D), MS (B, E), and Caps2-/- (C, F) mice. White dotted lines indicate the borders between the SuM and medial mammillary nucleus (MM). Scale bar = 50 μm. G, H, GPR54⁺ cell density in the SuMLL (G) and SuMRL (H). I, J, c-Fos⁺ cell density under the –social condition in the SuMLL (I) and SuMRL (J). K, L, c-Fos⁺ cell density under the +social condition in the SuMLL (K) and SuMRL (L). M, N, c-Fos⁺/GPR54⁺ cell density under the –social condition in the SuMLL (M) and SuMRL (N). O, P, c-Fos⁺/GPR54⁺ cell density under the +social condition in the SuMLL (O) and SuMRL (P). Q, R, Comparison of c-Fos⁺ cell density between the –social and +social conditions in the SuMLL (Q) and SuMRL (R). S, T, Comparison of c-Fos⁺/GPR54⁺ cell density between the –social and +social conditions in the SuMLL (S) and SuMRL (T). n = 3 sections per mouse, 3 mice per group. Filled circles represent values from individual sections. Data are presented as mean ± SEM (G–T). *p < 0.05, **p < 0.01, ***p < 0.001, ****p < 0.0001; ns, not significant. See Figure 1-2 for detailed statistical information. Download Figure 8-3, TIF file. [file eneuro-13-ENEURO.0440-25.2026-s013.tif]
